# Repeatome Dynamics and Sex Chromosome Differentiation in the XY and XY_1_Y_2_ Systems of the Fish *Hoplias malabaricus* (Teleostei; Characiformes)

**DOI:** 10.3390/ijms26136039

**Published:** 2025-06-24

**Authors:** Mariannah Pravatti Barcellos de Oliveira, Geize Aparecida Deon, Francisco de Menezes Cavalcante Sassi, Fernando Henrique Santos de Souza, Caio Augusto Gomes Goes, Ricardo Utsunomia, Fábio Porto-Foresti, Jhon Alex Dziechciarz Vidal, Amanda Bueno da Silva, Tariq Ezaz, Thomas Liehr, Marcelo de Bello Cioffi

**Affiliations:** 1 Evolutionary Cytogenetics Laboratory, Department of Genetics and Evolution, Federal University of São Carlos, São Carlos 13565-905, Brazil; mariannah@estudante.ufscar.br (M.P.B.d.O.); geizeadeon@gmail.com (G.A.D.); francisco.sassi@hotmail.com (F.d.M.C.S.); fernando_hsouza@outlook.com.br (F.H.S.d.S.); jhonalex279@gmail.com (J.A.D.V.); mbcioffi@ufscar.br (M.d.B.C.); 2 Fish Genomics and Conservation Laboratory, Faculty of Sciences, São Paulo State University, Bauru 01049-010, Brazil; caio.goes@unesp.br (C.A.G.G.); ricardo.utsunomia@unesp.br (R.U.); fp.foresti@unesp.br (F.P.-F.); amanda.bueno-silva@unesp.br (A.B.d.S.); 3 Institute of Applied Ecology, Faculty of Science and Technology, University of Canberra, Canberra 2617, Australia; tariq.ezaz@canberra.edu.au; 4 Institut für Humangenetik, Universitätsklinikum Jena, 07747 Jena, Germany

**Keywords:** single nucleotide polymorphism, repeatome, multiple sex systems

## Abstract

The wolf fish *Hoplias malabaricus* is a Neotropical species characterized by remarkable karyotypic diversity, including seven karyomorphs (KarA-G) with distinct sex chromosome systems. This study investigated the homologous XY (KarF) and XY_1_Y_2_ (KarG) sex chromosome systems present in this species by integrating cytogenetics and genomics to examine sex chromosomes’ composition through characterization of repeatome (satellite DNA and transposable elements) and sex-linked markers. Our analysis indicated that both karyomorphs are little differentiated in their sex chromosomes content revealed by satDNA mapping and putative sex-linked markers. Both repeatomes were mostly composed of transposable elements, but neither intra- (male versus female) nor interspecific (KarF x KarG) variations were found. In both systems, we demonstrated the occurrence of sex-specific sequences probably located on the non-recombining region of the Y chromosome supported by the accumulation of sex-specific haplotypes of HmfSat10-28/HmgSat31-28. This investigation offered valuable insights by highlighting the composition of homologous XY and XY_1_Y_2_ multiple sex chromosomes. Although homologous, the large Y chromosome in KarF corresponds to two separate linkage groups (Y_1_ and Y_2_) in KarG implying a specific meiotic arrangement involving the X chromosome in a meiotic trivalent chain. This scenario likely influenced recombination rates and, as a result, the genomic composition of these chromosomes.

## 1. Introduction

Over half of the existing vertebrate biodiversity consists of Teleostei fishes [1,2], which makes them a particularly appealing group for research across various evolutionary topics, including genomes and sex chromosome evolution [3]. Fish sex chromosomes are highly variable, unlike the largely conserved and heteromorphic features of sex chromosomes found in most mammals, birds, and snakes. These variations reflect different stages of evolutionary degeneration and differentiation [4,5]. Despite only 5% of studied teleost species displaying heteromorphic sex chromosomes, both female (ZW) and male (XY) heterogametic systems are present, alongside various multiple sex chromosome types, such as X_1_X_1_X_2_X_2_/X_1_X_2_Y, XX/XY_1_Y_2_, X_1_X_1_X_2_X_2_/X_1_Y_1_X_2_Y_2_, ZZ/ZW_1_W_2_, and Z_1_Z_1_Z_2_Z_2_/Z_1_W_1_Z_2_W_2_ [3].

**Figure 1:**
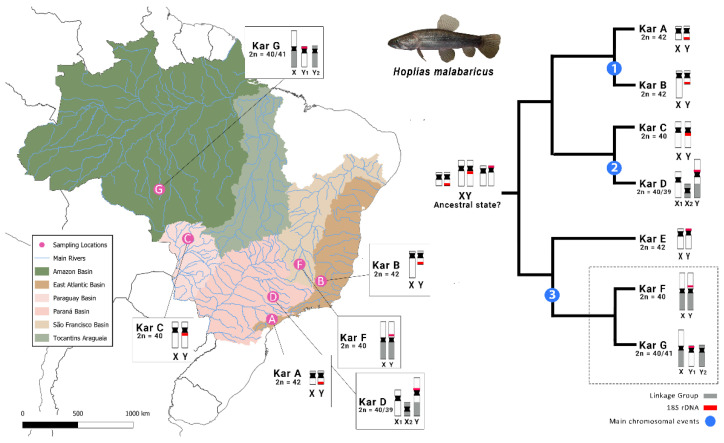
Geographic distribution and phylogenetic relationships of *H. malabaricus* karyomorphs. The map on the left illustrates the hydrographic basins in Brazil, indicating the karyomorph sampling locations in pink circles. The boxes highlight the corresponding karyomorphs, their distinct 2n, and sex chromosome morphologies. On the right, a phylogenetic tree proposes the evolutionary trajectory of the sex chromosome systems in these karyomorphs based on [8], with blue circles denoting key chromosomal events, following: (1) expansion of the X chromosome by repetitive DNA accumulation [7,9]; (2) sex chromosome–autosome translocation leading to multiple sex chromosomes [6,7]; and (3) conservation of the ancestral rearrangement (sex chromosome–autosome fusion) in a heterozygous condition resulting in multiple XX/XY_1_Y_2_ sex systems [10]. Legend on the lower right corner indicates the colors used in the idiograms for linkage groups and ribosomal DNA distribution, so as the main chromosomal events at the phylogeny branches.

The Neotropical fishes from the family Erythrinidae (Teleostei: Characiformes) are represented by three genera (*Erythrinus* Scopoli 1777, *Hoplias* Gill 1903, and *Hoplerythrinus* Gill 1895), with at least 17 valid species [2]. One of its most iconic representatives, the wolf fish *Hoplias malabaricus* exhibits a variety of karyotype diversity widespread in seven distinct karyomorphs, named KarA-G [6]. These karyomorphs are distinguished by differences in their diploid number (2n), chromosome sizes, morphology, and the presence of distinct sex chromosome systems (Figure 1). Five out of seven karyomorphs (B, C, D, F, and G) described so far display morphologically or molecularly differentiated sex chromosomes [6,7]. They include homomorphic XY systems (KarC and KarF); a heteromorphic XY system (KarB); and multiple sex chromosome systems (X_1_X_2_Y in KarD and XY_1_Y_2_ in KarG) (Figure 1).

This study focused on KarF and KarG, which underwent a parallel differentiation from a common ancestor (i.e., putatively similar to KarE) [10] (Figure 1). Conventional and molecular cytogenetic investigations indicate that in KarF, both the X and Y chromosomes fused with an autosomal pair, leading to the formation of a large metacentric XY pair. On the contrary, the KarG maintained this rearrangement in a heterozygous state, creating a large metacentric X chromosome, while the unfused homologs are segregated as the male-exclusive Y_1_ and Y_2_ chromosomes [10]. The KarF presents the same diploid number 2n = 40 in both sexes, with a male-specific region highlighted as a prominent interstitial heterochromatic block on the large metacentric Y chromosome, aligning with a series of microsatellite motifs and retrotransposons [10,11]. Meanwhile, the KarG presents 2n = 40 in females and 2n = 41 in males due to its XY_1_Y_2_ multiple sex chromosome system [6]. These karyomorphs are particularly compelling for investigation owing to their distinctive and divergent sex chromosomal systems, despite their common ancestry.

The integration of the cytogenetic and genomic fields proposed as “chromosomics” by [12] stands out as one of the most promising approaches for genome evolution studies. Indeed, the comparative analysis of repetitive DNA markers, like satellite DNAs (satDNAs) and transposable elements (TEs), has proven to be highly informative, particularly in the study of sex chromosome evolution [13,14,15,16]. In this context, numerous studies have examined heteromorphic XY or ZW systems focusing on satellite DNAs, as seen in *Triportheus* [15], *Megaleporinus* [17], and *Clarias* [18], as well as in transposable elements content in *Oncorhynchus* and *Salmo* [19], *Megaleporinus* and *Leporinus* [20], and *Apareiodon* [21]. Those have revealed that specific families of repetitive DNA may preferentially accumulate on sex-specific chromosomes (Y or W), suggesting their crucial role in chromosomal differentiation. Alongside repetitive DNA investigations, the examination of single nucleotide polymorphism (SNP) segregation in F1 progeny and its association with the heterogametic sex has facilitated the discovery of minor sex-linked regions across several species using cutting-edge genomic methodologies [22].

In fact, despite the identification of several sex chromosomes in fishes, most remain unexplored via comparative investigations. The effort to search for fish sex-linked sequences has intensified over the past decade, driven by the rapid advancement of high-throughput sequencing technologies such as restriction site-associated DNA sequencing (RADseq), which identifies polymorphic variants adjacent to specific restriction enzyme recognition sites [23]. The RAD-seq method was successfully applied to the identification of sex-linked regions in *Danio rerio* (zebrafish) [24], *Oreochromis niloticus* (Nile tilapia) [25], *Hippoglossus hippoglossus* (Atlantic halibut) [26], and *Dicentrarchus labrax* (European sea bass) [27]. RADseq has shown efficacy in identifying SNPs across diverse plant species, irrespective of reference genome availability [28]. This approach found 33,757 SNPs in *Pistacia vera* L., including all 38 putative sex-associated loci exhibiting female heterogamety [29]. Similarly, DArT-Seq (Diversity Arrays Technology, Canberra, Australia) is a proprietary sequencing pipeline that resembles ddRADseq by using a combination of restriction enzymes to produce fragments of hypomethylated regions, typically enriched with non-repetitive sequences [30]. The enhancement of active genomic regions via this methodology is beneficial for functional studies concentrating on expressed regions [31,32]. Given the anticipation of underexpression in certain sex-specific regions, DArT-seq presents a compelling method for identifying markers [33].

Sex chromosomes derive from a pair of autosomes, irrespective of their ancestral origins, and usually evolve in a canonical one-way direction of evolution [34,35]. However, despite significant progress in the study of sex chromosomes, little is known about the precise mechanisms underpinning their differentiation, especially those that took place in multiple sex chromosome systems. Aiming to provide insights on sex chromosome origin, differentiation, and composition, we have conducted a comparative analysis in two evolutionarily related sex chromosome systems: an early differentiated XY (KarF) and a multiple XY_1_Y_2_ (KarG) of the wolf fish *H. malabaricus*. We combined low-coverage whole genome sequencing techniques to describe the repeatome of both karyomorphs (particularly transposable elements and satellite DNA sequences), and cytogenetic in situ hybridization analysis of satDNAs, together with DArT-Seq to uncover putative sex-linked markers, to provide a comprehensive view on the evolution of these related sex chromosome systems.

## 2. Results

### 2.1. Satellitome Characterization

We determined that KarF and KarG each had a total of 56 and 45 satellite DNAs, respectively. KarF had satDNAs that were noticeably longer and displayed a higher number of satDNAs with varying abundances compared to KarG (11 satDNAs in KarF compared to only 8 satDNAs in KarG). Table 1 represents a compilation of the most noteworthy features of both satellitomes, and Tables S1 and S2 provide the full results.

The examination of satDNA library overlap between KarF and KarG demonstrated a significant similarity among different satellite families. Eleven satDNA families exhibited 100% sequence similarity, such as HmfSat10-28 and HmgSat31-28. Furthermore, many families had significant similarity without being entirely identical, such as HmfSat02-1894 and HmgSat10-705, which revealed a similarity of 74.51%, and HmfSat16-702 and HmgSat15-1140, which displayed the lowest similarity of 67.38%. In general, most satDNA families exhibited significant overlap, indicating that 36 satDNA families are common to both karyomorphs. Table S3 provides comprehensive details about their relationship, while Figures S1–S4 illustrate the alignment of all satellite DNA sequences.

### 2.2. Chromosomal Distribution of HmfSatDNAs and HmgSatDNAs

**Figure 2:**
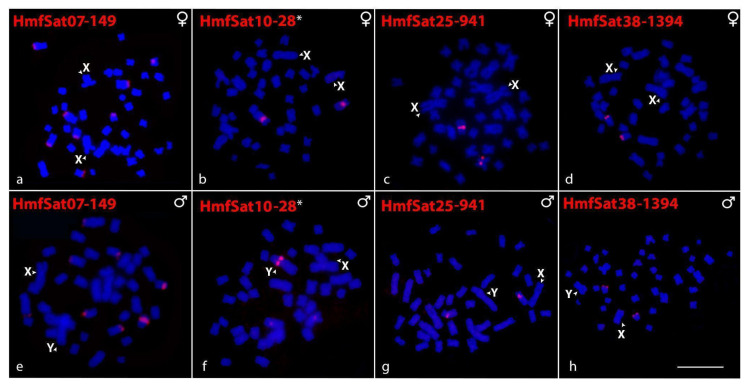
Female (**a**–**d**) and male (**e**–**h**) metaphase plates of KarF of *H. malabaricus* highlighting the chromosomal location of HmfSatDNAs. While HmfSat07-149, HmfSat25-941, and HmfSat38-1394 showed positive hybridization signals in autosomes, HmfSat10-28 was clustered in the Y chromosome (**f**). The satDNA family names are indicated on the left top, in red (Atto550-dUTP-labeled). The asterisk symbol (*) denotes that HmfSat10-28 and HmgSat31-28 exhibit 100% homology. Bar = 5 μm.

In KarF, six out of ten HmfSatDNAs have been successfully amplified. HmfSat07-149 (Figure 2a,e), HmfSat25-941 (Figure 2c,g), and HmfSat38-1394 (Figure 2d,h) all showed positive hybridization signals, exclusively localizing to autosomes and displaying similar distribution patterns in both sexes. In contrast, HmfSat10-28 was detected in a pair of autosomes (in both sexes) and on the Y chromosome (Figure 2b,f). The sequential C-banding to identify the Y sex chromosome in each HmfSatDNA hybridization experiment is arranged in Figure S5a–d. A complete list of primers used for amplification of probes is presented in Supplementary Table S4.

**Figure 3:**
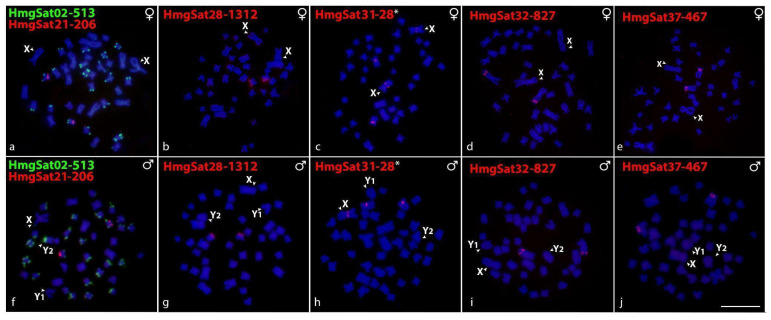
Female (**a**–**e**) and male (**f**–**j**) metaphase plates of KarG of *H. malabaricus* highlighting the chromosomal location of HmgSatDNAs. All HmgSatDNAs showed positive hybridization signals in the autosomes. Additionally, HmgSat02-513 was found in the Y_2_ (**f**) and HmgSat31-28 (HmfSat10-28) found in the X (**h**). The satDNA family names are indicated on the top left, in green (Atto488-dUTP-labeled) or red (Atto550-dUTP-labeled). The asterisk symbol (*) denotes that HmgSat31-28 and HmfSat10-28 exhibit 100% homology. Bar = 5 μm.

Five out of six HmgSatDNAs in the KarG were successfully amplified and produced positive results in hybridization assays: HmgSat21-206 (Figure 3a,f), HmgSat28-1312 (Figure 3b,g), HmgSat32-827 (Figure 3d,i), and HmgSat37-467 (Figure 3e,j) were all exclusively located in autosomes and displayed identical distribution patterns in both sexes. Conversely, HmgSat02-513 was mapped in nine pairs of autosomes and the centromeric region of the Y_2_ chromosome (Figure 3a,f). The HmfSat10-28 (HmgSat31-28), which exhibited positive signals on the Y chromosome of KarF, was hybridized in KarG and mapped in both X chromosomes (female) and one X chromosome (male), in addition to a pair of autosomes (Figure 3c,h). Figure S5e–i arranges the sequential whole-chromosome painting (WCP) to identify the sex chromosomes in each HmgSatDNA hybridization experiment.

### 2.3. Minimum Spanning Tree (MST)

**Figure 4:**
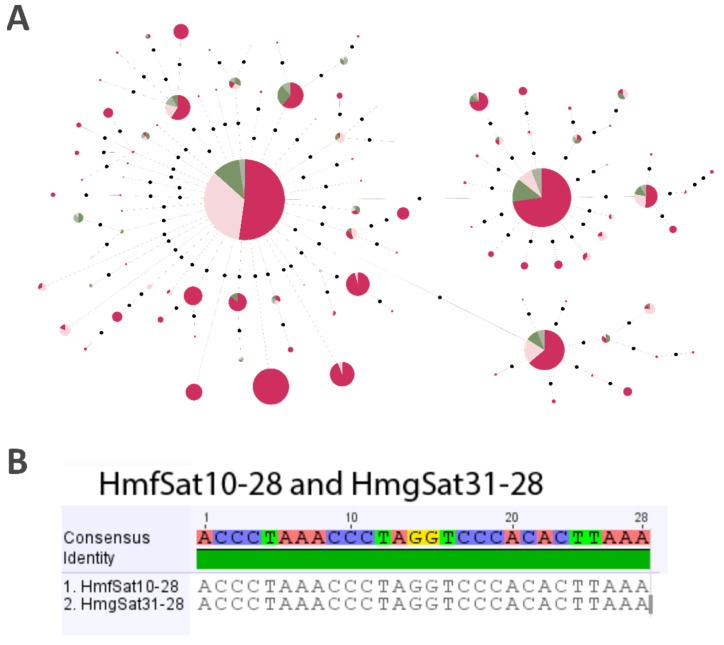
(**A**) Linear MST of HmfSat10-28 obtained from karyomorphs F (pink) and G (green) reads. Males are represented in dark green and dark pink, and females are represented in light green and light pink. Three major haplotypes were shared by both sexes of the two karyomorphs. Additionally, several abundant haplotypes were exclusively identified in KarF, indicating accumulation on the Y chromosome and lack of recombination with the X chromosome. Black dots indicate one nucleotide mutational step between each haplotype. (**B**) Alignment between HmfSat10-28 and HmgSat31-28.

We selected HmfSat10-28 to construct an MST from the male and female haplotypes of both karyomorphs. We selected this satDNA due to its location on the Y chromosome of KarF (Figure 2f) and the X chromosomes of KarG (Figure 3h), along with an autosomal pair in both karyomorphs. The MST of HmfSat10-28 indicated three prevalent haplotypes, particularly those associated with KarF, where this satDNA is more abundant (Figure 4). The three major haplotypes were common to both males and females of the two karyomorphs, owing to the presence of this satDNA in an autosomal pair. Nevertheless, numerous haplotypes exhibiting considerable abundance were exclusively identified in the KarF, indicating the accumulation of this sequence on the Y chromosome and implying an absence of recombination with the X chromosome (Figure 4).

### 2.4. Repeatome Composition of H. malabaricus Karyomorphs F and G

**Figure 5:**
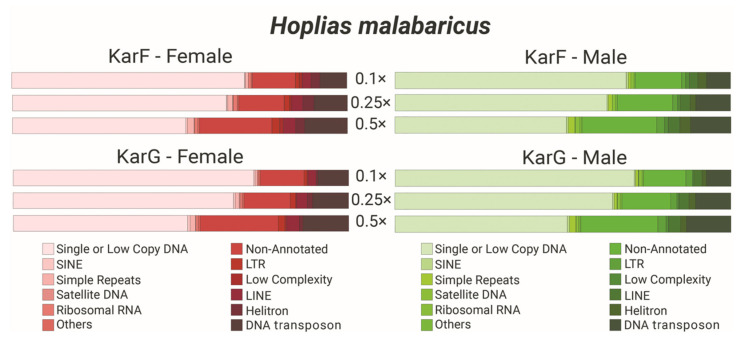
Analysis of the DNApipeTE repeatome content in both karyomorphs (F and G) of *Hoplias malabaricus*. Distinct coverages of 0.1, 0.25, and 0.5× were employed for each karyomorph. The legend delineates the color scheme for each repetition class. The comprehensive results are provided in Table S4.

The average total of repetitive sequences identified in both karyomorphs was approximately 34% and 38% for KarF (males and females, respectively) and about 37% for both sexes of KarG. The majority of the repetitive sequences were classified as non-annotated (Figure 5, Table S4), potentially indicating sequences unique to this species, as we utilized the DNApipeTE default repeat library without fish-specific annotations. DNA transposons constituted the most prevalent repetitive class in both karyomorphs, with a special participation of Helitron elements. Following, retrotransposons are present, including long terminal repeats (LTRs) and a higher content of long interspersed nuclear elements (LINEs) when compared to the short interspersed nuclear elements (SINEs). The detailed results are provided in Table S4. The comparisons of both sexes in each karyomorph or between karyomorphs revealed highly similar proportions, since no statistical differences were detected by the *t*-test (Table S5).

### 2.5. Sex-Linked Markers

We uncovered 50 putative sex-linked markers for KarG, indicating the occurrence of a male-heterogametic sex chromosome system (XY), while KarF did not present sex-linked markers. The statistical analysis suggested for DArTseq sex-linked markers [33] indicates that our data could present up to 0.189 and 96 sex-linked markers by chance in karyomorphs F and G, respectively. Consequently, although 50 potential sex-linked markers were identified in the KarG data, there is no statistical evidence to confirm that these markers are genuinely sex-linked rather than being found by chance. All the results, including the analysis through BLAST-2.16.0+, are provided in Table S6.

## 3. Discussion

Several investigations have been undertaken throughout the years to look at the *Hoplias* group and the evolution of its sex chromosome systems [6,7,8,10,11,36,37,38,39]. To contrast the distinct modes of evolution that might occur in simple and multiple systems, we concentrated on the XY and XY_1_Y_2_ sex chromosomes that are present in two of its karyomorphs (KarF and KarG, respectively). We focused on analyzing the repeatome composition of both karyomorphs and on uncovering possible sex-linked markers through complexity-reduction sequencing. The majority of both repeatomes consisted of TEs (primarily DNA transposons); however, neither intra- (male versus female) nor interspecific (KarF x KarG) variations were detected (Table S5). Similarly, the results indicated that KarF and KarG shared several satDNAs between them, but there are specific satDNAs for each karyomorph (Table S3). Moreover, a higher number of candidate SLMs and sex-linked satDNAs are present in KarG compared to KarF, underscoring the contrasting tempo and mode of evolution undertaken by simple and multiple sex chromosome systems (Table S6). Regrettably, KarG is a rare karyomorph with a documented distribution limited to a single locality (Aripuanã River—Mato Grosso state, Brazil); consequently, despite our efforts in sampling, this research is constrained in increasing the number of sampled individuals. This impairment hinders the acquisition of reliable sex-linked markers (please see methods). However, the vast majority of putative identified SLMs point to a male heterogametic sex chromosome system (XY-based), corroborated by our cytogenetic data. Given that the XY_1_Y_2_ represents an uncommon multiple sex chromosome system in fish [3], while the findings and discussion concerning the potential SLMs could be enhanced with broader sampling, they nonetheless provide significant insights into the composition and differentiation of this rare sex chromosome system.

### 3.1. Different Evolutionary Paths of Related Sex Chromosome Systems

*Hoplias malabaricus* is a model for evolutionary cytogenomic studies, especially regarding sex chromosomes, which are present in distinct stages of differentiation (i.e., homomorphic, heteromorphic, and multiple sex chromosome systems), with unique evolutionary pathways [6].

We focused on two distinct sex chromosome systems that share the same ancestral karyotype [10]. KarF has an early-differentiated XY sex chromosome system, with the Y chromosome in the nascent phases of differentiation [11]. This Y chromosome contains an interstitial heterochromatic male-specific region that accumulates the microsatellite motifs (A)n, (CAT)n, (CAC)n, (CGG)n, and (GAA)n, along with the LTR retrotransposon Rex1 [11,40], and distinct satDNA families, such as HmfSat10-28 (present study). On the other hand, a rare XY_1_Y_2_ multiple sex chromosome system is found in KarG, distinguished by the comparative genomic hybridization (CGH) between males and females, along with the distinct 2n by the two sexes, or by WCP [10]. This wide occurrence of sex chromosome systems, along with our findings regarding SLMs and satDNAs, lines up with the established canonical model of sex chromosome evolution. It posits that sex chromosomes originate from a pair of autosomes that stop recombining, leading to an accumulation of repetitive elements and sex-linked genes in the non-recombining regions. This ultimately results in the genetic and morphological differentiation of the sex chromosomes [41,42].

The common origin between the KarF and KarG is reinforced by the comparison of repeatomes, where the composition of TEs was similar between both karyomorphs (Table S5). On the other hand, although satellitomes were shared with a high degree of similarity between the karyomorphs (Table S3), certain sequences were unique to each species. Specific satDNAs that are more accumulated in males or females also differ between the karyomorphs, indicating that distinct amplification events of satDNA sequences occurred independently in each species due to their karyomorph-specific sex chromosome evolution, reinforcing the proposal that each karyomorph in *H. malabaricus* corresponds to independent evolutionary units [8].

The distribution of homologous satDNA also reflects this independent evolution of sex chromosomes. The HmfSat10-28, which presents 100% homology with HmgSat31-28, was identified on the X rather than in both Y chromosomes on KarG (Figure 3c,h). WCP experiments using HMF-Y (Y chromosome of KarF) and HMG-X/HMG-Y1 probes (X and Y_1_ chromosomes of KarG, respectively) have confirmed the homology between these chromosomes [10]; however, the two karyomorphs likely underwent distinct evolutionary trajectories. Our results show that the HmfSat10-28 in KarF (Y-distributed), have male-specific haplotypes, suggesting a contribution to the Y differentiation and indicating that it is putatively located in the non-recombining region. Conversely, its arrangement in KarG (X chromosome) may suggest that this satDNA precedes the degeneration of sex chromosomes and the formation of the non-recombining region, supporting the hypothesized creation of this multiple-sex chromosome system via Y fission [10]. Notably, a pair of autosomes was also detected by FISH with the HmfSat10-28/HmgSat31-28, revealing that most of the shared haplotypes indicated in Figure 4 might correspond to the autosomal variants. The MST analysis of HmfSat10-28 (Figure 4) indicated an abundance of haplotypes in the Y chromosome of KarF (in a total of 1519 haplotypes, 500 were exclusive to *H. malabaricus* KarF males), implying that an important portion of haplotypes are located in the non-recombining region (likely those in a large cluster detected by FISH in Figure 2), while most are present in the autosomes (see the shared haplotypes in males and females in Figure 4), thereby illustrating the differentiation and subsequent degeneration of the Y chromosome. However, the mechanism by which the sequences spread from the autosomes to the sex chromosomes or vice versa remains unclear.

TEs may significantly contribute to the dispersal of sequences within the genome and the emergence of novel tandem repeats, since a large percentage of the *H. malabaricus* KarF and KarG genome is composed of TEs, mainly DNA transposons (Figure 5, Table S5). So through TE duplication followed by unequal crossing-over or repair mechanisms activated by the transposase, the TEs could contribute to the emergence of new tandem repeats [43,44]. This evidence indicates that these sequences may play an important role in the genome of *H. malabaricus*, potentially contributing to the formation of novel satDNA sequences and to their dispersion on the genome.

The HmgSat02-513, located on many autosomal pairs, was further found in the terminal region of the Y2 sex chromosome of KarG (Figure 3f), which is proposed to have an autosomal origin by WCP experiments [10]. This configuration is atypical for satellite distribution in multiple sex chromosome systems, as, following the establishment of the heterozygous form of the rearrangement in KarG that resulted in the emergence of Y_1_ and Y_2_, only a limited number of sequences amplified their clusters. Comparatively, a satellitome analysis of the XY_1_Y_2_ system in the catfish *Harttia carvalhoi* has revealed three distinct patterns of accumulation: (i) sequences present on the X chromosome and retained on both Y_1_ and Y_2_; (ii) sequences found on the X and Y_2_, but absent in the Y_1_; and (iii) sequences exclusively located on the X chromosome, with no presence in the Y_1_ and Y_2_ [45], potentially reflecting common patterns seen in this rare multiple sex chromosome system. This configuration may indicate that HmgSat02-513 either dispersed after the chromosomal rearrangement associated with the emergence of this system or that it was present on the autosome prior to the rearrangement but is located in the non-recombining region, thereby preventing its transmission to the other sex chromosomes. When treating the Y_1_ and Y_2_ chromosomes as independently evolving entities, the probability of the emergence of chromosome-specific sex sequences rises, thereby promoting the differentiation of the sex chromosomes within these karyomorphs. Disparities between the satDNA catalogs of males and females are also evident in the percentage contribution of these elements to the genome. The satMiner protocol utilized in this study for constructing the satDNA library indicates that these elements constitute 6.1% (KarF) and 7.7% (KarG) of male genomes, whereas female genomes comprise 5.3% (KarF) and 7% (KarG) of satDNAs. Despite being low, this variation between sexes may be attributable to sex-specific haplotypes accumulated in the sex chromosomes. Indeed, the low degree of difference between males and females is expected, since the early evolved and/or multiple sex chromosomes usually have a high recombination rate, avoiding the accumulation of deleterious sequences in heterogametic chromosomes [revised in 41]. The repetitive DNA annotation performed in DNApipeTE identified particular patterns, with satDNAs comprising 0.15% in KarF males, 0.1% in KarF females, 0.13% in KarG males, and 0.14% in KarG females. The reduced amount of satDNAs generated by DNApipeTE, compared to the results from satMiner (TAREAN), was expected, since the latter applied a successive iteration strategy to uncover satDNA content, thus providing a more thorough methodology for constructing satDNA libraries [13].

### 3.2. Insights on Sex Chromosome Differentiation Through Repetitive DNAs and Putative Sex-Linked Markers

Following earlier ddRAD methods, we used DArTseq (Diversity Arrays Technology), which combines reducing genome complexity with restriction enzymes (one that cuts frequently and another that targets less methylated areas) and next-generation sequencing to create high-quality markers. Lambert et al. [33] highlight that DArTseq is a useful method for finding sex-linked markers in species that are not commonly studied, like *H. malabaricus*, because it is less costly, easier, and more reliable than other methods such as RFLP and AFLP [46]. Consequently, it has been effectively used in several species, encompassing plants [47,48] and animal species [33,49], precisely finding loci associated with sex chromosomes and demonstrating effectiveness in elucidating sex-determination mechanisms.

Our findings indicate that KarG displayed greater satDNA accumulation in its sex chromosomes and higher levels of potential SLM in comparison to KarF (Table S7); however, the results on this matter lack statistical confidence to assess if the markers are truly sex-linked ones (please see the Section 2). The observed discrepancies with satDNAs may suggest that evolutionary processes operated in KarG for longer periods or at a higher rate, resulting in the accumulation of repetitive sequences. This accumulation could have contributed to the differentiation of its sex chromosomes. Genetic drift may also contribute by randomly establishing certain variations throughout the population over time [50], resulting in the accumulation of both repetitive sequences and putative SLM.

We selected the putative SLM uncovered by DArT-sex for BLAST searches against the NCBI non-redundant nucleotide collection to seek evidence of sex linkage (i.e., sequences situated on sex chromosomes or sex-related genes). The results indicated sequences linked to various biological processes, such as regulation, transport, RNA processing, hormone reception, and enzyme activity; however, with the exception of Regulatory Factor X4 (RFX4), no findings demonstrated a definitive relationship between the SLM and a sex chromosome (Table S6).

Among our BLAST matches, RFX4 is indeed the most intriguing discovery. Transcription factors (TFs) possess a conserved DNA-binding domain (DBD) of the winged-helix type, allowing them to control several genes [51]. These RFX have shown essential functions in several species by regulating fundamental processes like cell cycle progression, DNA repair, and cellular differentiation [52,53,54]. This gene is shown to affect swim bladder inflation and shape, along with body growth and spinal curvature in several fish groups [55], while also being crucial for human testis development [56]. RFX4, sometimes referred to as testis development protein NYD-SP10, has been implicated in the regulation of spermatogenesis and male sexual development, as shown by prior research [57], and may contribute to sex chromosome differentiation. Regrettably, a chromosome-level genome assembly for *H. malabaricus* karyomorphs F or G was unavailable during this study; hence, we were unable to link this sequence to the genome to confirm its position on the sex chromosomes.

The striking similarity of repetitive DNA catalogs observed in both DNApipeTE results and the satDNA libraries suggests that the major rearrangement event involving the Y chromosome in KarG did not result in significant alterations to its repetitive DNA composition. The unique arrangement of certain satDNAs, like HmgSat02-513, shown by FISH, indicates that more changes happened after the original Y chromosome split into the Y and Y in KarG. Indeed, previous repetitive DNA FISH mapping using probes from microsatellites and the 5S rDNA [10,11] indicates a differential distribution of repetitive DNA sequences at the Y_1_ and Y_2_ of KarG, which does not occur in the metacentric Y of KarF. When the proposed rearrangement of the ancestral XY of both karyomorphs occurred, the Y from KarF was turned into a single linkage group, forming a large metacentric chromosome, while in KarG the Y_1_ remained as an acrocentric chromosome and the Y_2_ in a submetacentric chromosome [10]. In this way, with an exception for the pseudoautosomal region that both Y_1_ and Y_2_ have with the X, the male-specific chromosomes can present an independent arrangement of repetitive sequences without an impairment of their pairing with the X. The only condition for this independent accumulation of repetitive sequences in Y_1_ and Y_2_ in KarG when compared with its homologous Y of KarF is that it has occurred after the rearrangement and the establishment of the karyomorphs; otherwise, it would be visualized also in KarF. The canonical model of sex chromosome evolution, as reviewed in [58], states that multiple sex chromosomes emerge following the establishment of the non-recombining region, during the degeneration process, and either subsequent to rearrangements with autosomes or through fissions/fusions of the ancestral sexual pair. In *H. malabaricus*, the proposed mechanism for the emergence of the homologous XY and XY_1_Y_2_ observed in KarF and KarG, respectively, likely did not involve a turnover of the sex determination region. The disparities in the chromosomal distribution of repetitive sequences may be associated with the pseudoautosomal region, which functions like autosomes during crossing-over, facilitating the exchange of sequences between sex chromosomes and potentially other autosomes, as previously demonstrated for human chromosomes of analogous shape and size [59]. Furthermore, the existence of two Y chromosomes in KarG establishes a novel and partially autonomous component implicated in meiotic exchange, which elucidates the unique distribution of satDNAs noted in each karyomorph.

Although homologous, the fact that the large Y chromosome in KarF corresponds to two separate linkage groups (Y_1_ and Y_2_) in KarG implies a specific meiotic arrangement involving the X chromosome in a meiotic trivalent chain. This scenario probably influenced recombination rates and, as a result, the genomic composition of these chromosomes, as herein indicated by the variations observed related to satDNAs, potential sex-linked SNPs, and other classes of repetitive DNA. Additional research involving other *H. malabaricus* karyomorphs with standard (KarA, B, C, E) and multiple (KarD) sex chromosome systems may yield additional information needed to clarify this concept. Further investigations also require an expanded sampling size on each karyomorph to minimize potential batch effects created during sequencing and to allow deep comparisons among karyomorphs and sexes.

## 4. Materials and Methods

### 4.1. Chromosome Preparation, DNA Extraction, and Low-Coverage Genome Sequencing

Fifteen individuals (seven males and eight females) of karyomorph F and six individuals (three males and three females) of karyomorph G of *H. malabaricus* were analyzed in this study: KarF (XY sex system) from São Francisco River, Minas Gerais state (18°31′26.9″ S / 45°14′5.7″ W) and KarG (XY_1_Y_2_) from Aripuanã River, Mato Grosso state (10°45′12.2″ S / 59°15′34.8″ W), both in Brazil. The specimens were collected from wild populations with authorization from the National System for the Management of Genetic Heritage and Associated Traditional Knowledge (SISGEN-A96FF09), the Chico Mendes Institute for Biodiversity Conservation (ICMBIO), and the System of Authorization and Information about Biodiversity (SISBIO) under license numbers 10538-3 and 15117-1. The experiments followed the ethical standards set by the Federal University of São Carlos Ethics Committee on Animal Experimentation (CEUA) under process number 7994170423.

Mitotic chromosomes were obtained from kidney cells according to Bertollo et al. [60]. The specimens were treated with a 0.005% colchicine solution for 30 min, followed by anterior kidney extraction and hypotonic treatment with 0.075M KCl. The tissue was carefully fragmented to obtain a homogeneous cell suspension, incubated at 37 °C for 20 min, and fixed with methanol-acetic acid (3:1). After three fixation cycles, the final suspension was stored at −20 °C. Additionally, liver samples were used to extract the genomic DNAs (gDNAs) following the phenol-chloroform method according to Sambrook and Russell [61]. Each sample was processed separately, and one male and one female sample were sequenced utilizing the BGISEQ-500 platform (paired-end 2× 150 bp). The obtained reads were deposited in the Sequence Read Archive (SRA-NCBI) and are available under accession numbers SRR31316061 (KarF female); SRR31316062 (KarF male); SRR31316059 (KarG female); and SRR31316060 (KarG male).

### 4.2. Characterization of the Satellitome

Initially, we performed quality and adapter trimming using Trimmomatic v. 0.33 [62] for each library. After that, we used Tandem Repeat Analyzer (TAREAN) (Galaxy Version 2.3.12.1) [63] to characterize their satellitomes, using 2 × 500,000 reads in each iteration. Tandem sequences were filtered using DeconSeq (version 0.4.3) [64], and the process was repeated until no low- or high-confidence satDNA remained. Then, we identified and removed other usual output tandemly repeated sequences, such as multigene families (rDNAs and U snDNAs), from the catalog. Finally, we performed a similarity search with RepeatMasker (version 4.1.9) using a custom Python (version 3.12) script (accessed on 13 April 2024 https://github.com/fjruizruano/ngs-protocols/blob/master/rm_homology.py) to detect redundancies in the catalog and classify the isolated satDNAs as the same variant, different variants of the same family, or superfamilies (similarities greater than 95, 80, and 50%, respectively) [13]. The abundance of each satDNA was estimated with RepeatMasker [65], using a custom Python script (accessed on 17 April 2024 https://github.com/fjruizruano/ngs-protocols/blob/master/repeat_masker_run_big.py). We utilized 2 × 5,000,000 randomly selected reads, and genomic abundance was given as the number of mapped reads in each satDNA divided by the number of analyzed nucleotides. Kimura’s divergence was obtained with the Kimura 2-parameter model from the script calcDivergenceFromAlign.pl of the RepeatMasker suite [65]. The satDNAs were classified in decreasing abundance order and named according to [13], with the species abbreviation and the letter of each karyomorph (Hmf and Hmg), in addition to the term “Sat” and the catalog number. Considering the sex chromosome systems, we calculated the ratio between the abundance of each satDNA in male and female libraries to search for potential satDNAs that are accumulated in the sex chromosomes. The catalogs were deposited on GenBank (NCBI) with the accession numbers PQ062407-PQ062462 for KarF and PQ062463-PQ062507 for KarG.

We also extracted monomers from male and female genomic libraries of both karyomorphs to calculate abundance and monomer diversity scores for satDNAs accumulated in the sex chromosomes. We selected HmfSat10-28 due to its accumulation in chromosome Y in KarF and in the X in KarG (see results). For this, we collected monomers using a random subsample of 2 × 500,000 reads for each sample. Then, we aligned the isolated reads against the satDNA sequence to extract only the region corresponding to one monomer. After that, we discarded monomers found only once (singletons) with CD-HIT [66] to avoid sequencing errors. Finally, a minimum spanning tree (MST) was constructed using PHYLOVIZ (version 2.0) [67]. We used MegaBLAST [68] to compare the satDNA sequences with the NCBI collection.

### 4.3. Satellite Amplification, Labeling, and Fluorescence In Situ Hybridization (FISH)

Subsequent to the characterization of both satellitomes, we developed primers for satDNAs that shown relevant disparities in abundance between males and females in both karyomorphs. We developed primers for six HmgSatDNAs in KarG, while 10 primers were formulated for KarF HmfSatDNAs. These satDNAs were amplified using the conditions described by [15], and the resulting products were analyzed through a 2% agarose gel electrophoresis. For the labeling process, we used the Nick-Translation Labeling Kit (Jena Bioscience, Germany) with Atto488-dUTP (green) or Atto550-dUTP (red), according to the manufacturer’s instructions. For the HmfSatDNA10-28, which presented a low repeat unit length (RUL), we directly labeled it with Cy3 at the 5′ end during the synthesis by ThermoFisher (ThermoFisher Scientific—Waltham, MA, USA). Finally, the FISH procedures were conducted under high-stringency conditions, as described by [69].

To properly identify the sex chromosomes, we used two different strategies: for the KarF, C-positive heterochromatin was detected following the protocol described by [70]. This procedure allowed us to identify the Y chromosome by its distinctive interstitial C-positive band present on the long arms [11]. Conversely, identification of the sex chromosomes of KarG were performed using whole-chromosome painting probes (HMG-X and HMG-Y1), previously obtained by microdissection [10]. The FISH conditions employed were the same as those previously described.

### 4.4. Repeatome Analysis

To access the whole repetitive DNA composition (repeatome) of our samples, we conducted the DNApipeTE (version 1.4) pipeline, which is optimized for classifying and annotating repetitive DNA in low-coverage data (<1×) without requiring genome assembly [71]. The primary objective is to construct a representative repeat library while also detecting, quantifying, and estimating the relative abundance of transposable elements (TEs), thereby serving as a complementary approach to our RepeatExplorer2 analysis, which specializes in identifying satellite DNAs (satDNAs). We ran DNApipeTE on single-end forward reads for males and females of karyomorphs F and G. We used the reference genome size of *H. malabaricus* (1.2 Gb) (GCF_029633855.1) for all samples, assuming that genome size does not vary among the analyzed karyomorphs, and used as genome coverage (0.1×, 0.25× and 0.5×) and sample_number: 2. Note that this genome belongs to a distinct karyomorph and thus was not used for additional comparisons. We performed a paired *t*-test on R version 4.4.3 [72] to compare the male and female catalogs of each karyomorph. The entire catalogs were compared, as specific comparisons for each repeat class are indeterminate due to division by zero. We employed a 95% confidence threshold to identify significant variations.

### 4.5. Genotyping by Sequencing (GBS) and Sex-Linked Markers Analysis

The DNA extraction was performed on liver tissue of all sampled individuals from both karyomorphs. The DNA samples were sequenced on the Illumina HiSeq 2500 platform by DArT-Seq (Diversity Arrays Technology Pty Ltd., Canberra, Australia), a complexity reduction method that generates ~69 bp reads. All sequences were processed with the pyRAD v3.3.0.66 pipeline [73]. After trimming sequence adapters, reads were filtered by quality. Sequences with more than five undetermined bases or low-quality bases (Q < 33) were removed from the dataset. Filtered sequences were aligned and clustered for each individual to define the loci. Following the estimation of error rates, the mean frequencies of heterozygosity loci are clustered across individuals. Sequences shorter than 35 base pairs are removed, and a final dataset of sequences is generated. These data were created by [8] from the F1, F2, F3, and G1 sampling sites and are used here to find possible sex-linked markers (SLM) following the standard method for DArTseq data [33]. We first organized the columns in male and female groups, then used the “countif” function in Microsoft Excel v. 2505 (Office 365, Microsoft Corporation) to count all heterozygous results (2) for males or females, using as a counterpart the counting of homozygous results (either for the reference “0” or the SNP “1”). Those SNPs presenting a heterozygous state for all males and homozygous for all females were treated as indicative of a male-linked sex determination system (XY), whereas the inverse indicates a putative female-linked (ZW) sex determination system. To test the statistical significance of the obtained SLMs, we performed the calculation suggested by Lambert et al. [33]. This calculation estimates the number of SLMs found by mere chance, making it possible to assess if the dataset is suitable for the identification of SLMs. The putative sex-linked SNPs were then arranged in a “fasta” file and BLAST-searched against the NCBI collection to check for identity with sequences deposited in the database [68]. For this, we used Blast2GO [74] with the following parameters: blastn-short, e-value = 1 × 10^−5^, database = NR, and taxonomy filter = Actinopterygii. We also checked for possible homology between the SLM of each karyomorph using UGENE 50.0 [75]. Putative SLMs were mapped against the satDNA list obtained, but no positive matches were found.

## Figures and Tables

**Table 1:** Main satellitome characteristics of both *H. malabaricus* karyomorphs F and G, including the number of satellite DNA sequences (N), smallest and largest repeat unit length in bp (SmRUL and LgRUL, respectively), and approximate representation of the satDNAs in both male (M%) and female (F%) genomes obtained by the satMiner protocol.

Karyomorph	N	SmRUL	LgRUL	M%	F%
KarF	56	28 (HmfSat10)	2944 (HmfSat08)	6.1	5.3
KarG	45	21 (HmgSat41)	1380 (HmgSat27)	7.7	7

## Data Availability

The datasets generated and analyzed during the current study are available in the GenBank repository. The satellite DNA sequences are under the accession numbers PQ062407-PQ062462 for KarF and PQ062463-PQ062507 for KarG.

## Supplementary Materials

The following supporting information can be downloaded at: https://www.mdpi.com/article/10.3390/ijms26136039/s1.

## Abbreviations

The following abbreviations are used in this manuscript:

Kar	Karyomorph
2n	Diploid number
satDNA	Satellite DNA
TE	Transposable Element
SNP	Single Nucleotide Polymorphism
RADseq	Restriction Site-associated DNA Sequencing
ICMBIO	Chico Mendes Institute of Biodiversity Conservation
ISBIO	Brazilian Biodiversity Information and Authorization System
CEUA	Ethics Committee on Animal Experimentation
FISH	Fluorescence in situ Hybridization
DarT-Seq	Diversity Arrays Technology Sequencing
SLM	Sex-linked Marker
RFLP	Restriction Fragment Length Polymorphism
AFLP	Amplified Fragment Length Polymorphism
RFX4	Regulatory Factor X 4
TF	Transcription Factor
DBD	DNA-binding Domain

## References

[1] Nelson J.S., Grande T.C., Wilson M.V. (2016). Fishes of the World.

[2] Fricke R., Eschmeyer W.N., Fong J.D. (2024). Eschmeyer’s Catalog of Fishes: Genera/Species by Family/Subfamily. http://researcharchive.calacademy.org/research/ichthyology/catalog/SpeciesByFamily.asp.

[3] Sember A., Nguyen P., Perez M.F., Altmanová M., Ráb P., Cioffi M.D.B. (2021). Multiple sex chromosomes in teleost fishes from a cytogenetic perspective: State of the art and future challenges. Philos. Trans. R. Soc. Lond. B Biol. Sci..

[4] Graves J.A.M., Shetty S. (2001). Sex from W to Z: Evolution of vertebrate sex chromosomes and sex-determining genes. J. Exp. Zool..

[5] Schartl M., Schmid M., Nanda I. (2016). Dynamics of vertebrate sex chromosome evolution: From equal size to giants and dwarfs. Chromosoma.

[6] Bertollo L.A.C., Born G.G., Dergam J.A., Fenocchio A.S., Moreira-Filho O. (2000). A biodiversity approach in the neotropical Erythrinidae fish, *Hoplias malabaricus*: Karyotypic survey, geographic distribution of cytotypes, and cytotaxonomic considerations. Chromosome Res..

[7] Cioffi M.B., Martins C., Vicari M.R., Rebordinos L., Bertollo L.A.C. (2010). Differentiation of the XY sex chromosomes in the fish *Hoplias malabaricus* (Characiformes, Erythrinidae): Unusual accumulation of repetitive sequences on the X chromosome. Sex. Dev..

[8] Souza F.H.S.S., Perez M.F., Ferreira P.H., Bertollo L.A.C., Ezaz T., Charlesworth D., Cioffi M.B. (2024). Multiple karyotype differences between populations of the *Hoplias malabaricus* (Teleostei; Characiformes), a species complex in the gray area of the speciation process. Heredity.

[9] Born G.G., Bertollo L.A.C. (2000). An XX/XY sex chromosome system in a fish species, *Hoplias malabaricus*, with a polymorphic NOR-bearing X chromosome. Chromosome Res..

[10] de Oliveira E.A., Sember A., Bertollo L.A.C., Yano C.F., Ezaz T., Moreira-Filho O., Hatanaka T., Trifonov V., Liehr T., Al-Rikabi A.B.H. (2018). Tracking the evolutionary pathway of sex chromosomes among fishes: Characterizing the unique XX/XY_1_Y_2_ system in *Hoplias malabaricus* (Teleostei, Characiformes). Chromosoma.

[11] Freitas N.L., Al-Rikabi A.B., Bertollo L.A.C., Ezaz T., Yano C.F., de Oliveira E.A., Hatanaka T., Cioffi M.B. (2018). Early stages of XY sex chromosomes differentiation in the fish *Hoplias malabaricus* (Characiformes, Erythrinidae) revealed by DNA repeats accumulation. Curr. Genomics..

[12] Deakin J.E., Potter S., O’Neill R., Ruiz-Herrera A., Cioffi M.B., Eldridge M.D., Fukui K., Graves J.A.M., Griffin D., Grutzner F. (2019). Chromosomics: Bridging the gap between genomes and chromosomes. Genes.

[13] Ruiz-Ruano F.J., López-León M.D., Cabrero J., Camacho J.P.M. (2016). High-throughput analysis of the satellitome illuminates satellite DNA evolution. Sci. Rep..

[14] Utsunomia R., Silva D.M., Ruiz-Ruano F.J., Goes C.A.G., Melo S., Ramos L.P., Oliveira C., Porto-Foresti F., Foresti F., Hashimoto D.T. (2019). Satellitome landscape analysis of *Megaleporinus macrocephalus* (Teleostei, Anostomidae) reveals intense accumulation of satellite sequences on the heteromorphic sex chromosome. Sci. Rep..

[15] Kretschmer R., Goes C.A.G., Bertollo L.A.C., Ezaz T., Porto-Foresti F., Toma G.A., Utsunomia R., Cioffi M.B. (2022). Satellitome analysis illuminates the evolution of ZW sex chromosomes of Triportheidae fishes (Teleostei: Characiformes). Chromosoma.

[16] Goes C.A.G., Dos Santos R.Z., Aguiar W.R.C., Alves D.C., Silva D.M., Foresti F., Oliveira C., Utsunomia R., Porto-Foresti F. (2022). Revealing the satellite DNA history in *Psalidodon* and *Astyanax* characid fish by comparative satellitomics. Front. Genet..

[17] Crepaldi C., Martí E., Gonçalves E.M., Martí D.A., Parise-Maltempi P.P. (2021). Genomic differences between the sexes in a fish species seen through satellite DNAs. Front. Genet..

[18] Lisachov A., Nguyen D.H.M., Panthum T., Ahmad S.F., Singchat W., Ponjarat J., Jaisamut K., Srisapoome P., Duengkae P., Hatachote S. (2023). Emerging importance of bighead catfish (*Clarias macrocephalus*) and North African catfish (*C. gariepinus*) as a bioresource and their genomic perspective. Aquaculture.

[19] Faber-Hammond J.J., Phillips R.B., Brown K.H. (2015). Comparative Analysis of the Shared Sex-Determination Region (SDR) among Salmonid Fishes. Genome Bio. Evol..

[20] Crepaldi C., Cabral-de-Mello D.C., Parise-Maltempi P.P. (2024). Comparative analysis of transposable elements dynamics in fish with different sex chromosome systems. Genome.

[21] Schemberger M.O., Nascimento V.D., Coan R., Ramos É., Nogaroto V., Ziemniczak K., Valente G.T., Moreira-Filho O., Martins C., Vicari M.R. (2019). DNA transposon invasion and microsatellite accumulation guide W chromosome differentiation in a Neotropical fish genome. Chromosoma.

[22] Palmer D.H., Rogers T.F., Dean R., Wright A.E. (2019). How to identify sex chromosomes and their turnover. Mol. Ecol..

[23] Baird N.A., Etter P.D., Atwood T.S., Currey M.C., Shiver A.L., Lewis Z.A., Selker E.U., Cresko W.A., Johnson E.A. (2008). Rapid SNP discovery and genetic mapping using sequenced RAD markers. PLoS ONE.

[24] Anderson J.L., Rodríguez Marí A., Braasch I., Amores A., Hohenlohe P., Batzel P., Postlethwait J.H. (2012). Multiple sex-associated regions and a putative sex chromosome in zebrafish revealed by RAD mapping and population genomics. PLoS ONE.

[25] Palaiokostas C., Bekaert M., Khan M.G., Taggart J.B., Gharbi K., McAndrew B.J., Penman D.J. (2013). Mapping and validation of the major sex-determining region in Nile tilapia (*Oreochromis niloticus* L.) using RAD sequencing. PLoS ONE.

[26] Palaiokostas C., Bekaert M., Davie A., Cowan M.E., Oral M., Taggart J.B., Gharbi K., McAndrew B.J., Penman D.J., Migaud H. (2013). Mapping the sex determination locus in the Atlantic halibut (*Hippoglossus hippoglossus*) using RAD sequencing. BMC Genom..

[27] Palaiokostas C., Bekaert M., Taggart J.B., Gharbi K., McAndrew B.J., Chatain B., Penman D.J., Vandeputte M. (2015). A new SNP-based vision of the genetics of sex determination in European sea bass (*Dicentrarchus labrax*). Genet. Sel. Evol..

[28] Barchi L., Lanteri S., Portis E., Acquadro A., Valè G., Toppino L., Rotino G.L. (2011). Identification of SNP and SSR markers in eggplant using RAD tag sequencing. BMC Genom..

[29] Kafkas S., Khodaeiaminjan M., Güney M., Kafkas E. (2015). Identification of sex-linked SNP markers using RAD sequencing suggests ZW/ZZ sex determination in *Pistacia vera* L. BMC Genom..

[30] Kilian B., Graner A. (2012). NGS technologies for analyzing germplasm diversity in genebanks. Brief. Funct. Genom..

[31] Meyer K.D. (2019). DART-seq: An antibody-free method for global m6A detection. Nat. Methods.

[32] Pereira W.J., Pappas M.d.C.R., Grattapaglia D., Pappas G.J.J. (2020). A cost-effective approach to DNA methylation detection by Methyl Sensitive DArT sequencing. PLoS ONE.

[33] Lambert M.R., Skelly D.K., Ezaz T. (2016). Sex-linked markers in the North American green frog (*Rana clamitans*) developed using DArTseq provide early insight into sex chromosome evolution. BMC Genom..

[34] Ohno S., Muramoto J., Christian L., Atkin N.B. (1967). Diploid-tetraploid relationship among old-world members of the fish family Cyprinidae. Chromosoma.

[35] Charlesworth B., Crispin Y.J., Charlesworth D. (2014). The evolutionary dynamics of sexually antagonistic mutations in pseudoautosomal regions of sex chromosomes. Evolution.

[36] Bertollo L.A.C., Takahashi C.S., Moreira-Filho O. (1983). Multiple sex chromosomes in the genus *Hoplias* (Pisces: Erythrinidae). Cytologia.

[37] Cioffi M.B., Camacho J.P.M., Bertollo L.A.C. (2011). Repetitive DNAs and differentiation of sex chromosomes in neotropical fishes. Cytogenet. Genome Res..

[38] Cioffi M.B., Liehr T., Trifonov V., Molina W.F., Bertollo L.A.C. (2013). Independent sex chromosome evolution in lower vertebrates: A molecular cytogenetic overview in the Erythrinidae fish family. Cytogenet. Genome Res..

[39] Toma G.A., Sember A., Goes C.A.G., Kretschmer R., Porto-Foresti F., Bertollo L.A.C., Liehr T., Utsunomia R., Cioffi M.B. (2024). Satellite DNAs and the evolution of the multiple X_1_X_2_Y sex chromosomes in the wolf fish *Hoplias malabaricus* (Teleostei; Characiformes). Sci. Rep..

[40] Sember A., Bertollo L.A.C., Ráb P., Yano C.F., Hatanaka T., De Oliveira E.A., Cioffi M.B. (2018). Sex chromosome evolution and genomic divergence in the fish *Hoplias malabaricus* (Characiformes, Erythrinidae). Front. Genet..

[41] Charlesworth D. (2021). The timing of genetic degeneration of sex chromosomes. Philos. Trans. R. Soc. Lond. B Biol. Sci..

[42] Kratochvíl L., Stöck M., Rovatsos M., Bullejos M., Herpin A., Jeffries D.L., Peichel C.L., Perrin N., Valenzuela N., Pokorná M.J. (2021). Expanding the classical paradigm: What we have learnt from vertebrates about sex chromosome evolution. Philos. Trans. R. Soc. Lond. B Biol. Sci..

[43] Kapitonov V.V., Jurka J. (1999). The Long Terminal Repeat of an Endogenous Retrovirus Induces Alternative Splicing and Encodes an Additional Carboxy-Terminal Sequence in the Human Leptin Receptor. J. Mol. Evol..

[44] Wong L.H., Choo K.H.A. (2004). Evolutionary dynamics of transposable elements at the centromere. Trends Genet..

[45] Deon G.A., Dos Santos R.Z., Sassi F.D.M.C., Moreira-Filho O., Vicari M.R., Porto-Foresti F., Utsunomia R., Cioffi M.B. (2024). The role of satellite DNAs in the chromosomal rearrangements and the evolution of the rare XY_1_Y_2_ sex system in *Harttia* (Siluriformes: Loricariidae). J. Hered..

[46] Sopniewski J., Shams F., Scheele B.C., Kefford B.J., Ezaz T. (2019). Identifying sex-linked markers in *Litoria aurea*: A novel approach to understanding sex chromosome evolution in an amphibian. Sci. Rep..

[47] Gelaw Y.M., Eleblu J.S., Ofori K., Fenta B.A., Mukankusi C., Emam E.A., Offei S. (2023). High-density DArTSeq SNP markers revealed wide genetic diversity and structured population in common bean (*Phaseolus vulgaris* L.) germplasm in Ethiopia. Mol. Biol. Rep..

[48] Baloch F.S., Alsaleh A., Shahid M.Q., Çiftçi V., Sáenz de Miera L.E., Aasim M., Nadeem M.A., Aktaş H., Özkan H., Hatipoğlu R. (2017). A whole genome DArTseq and SNP analysis for genetic diversity assessment in durum wheat from central fertile crescent. PLoS ONE.

[49] Hill P.L., Burridge C.P., Ezaz T., Wapstra E. (2018). Conservation of sex-linked markers among conspecific populations of a viviparous skink, *Niveoscincus ocellatus*, exhibiting genetic and temperature-dependent sex determination. Genome Biol. Evol..

[50] Kimura M. (1955). Random Genetic Drift in Multi-Allelic Locus. Evolution.

[51] Gajiwala K.S., Chen H., Cornille F., Roques B.P., Reith W., Mach B., Burley S.K. (2000). Structure of the winged-helix protein hRFX1 reveals a new mode of DNA binding. Nature.

[52] Zaim J., Speina E., Kierzek A.M. (2005). Identification of new genes regulated by the Crt1 transcription factor, an effector of the DNA damage checkpoint pathway in *Saccharomyces cerevisiae*. J. Biol. Chem..

[53] Garg A., Futcher B., Leatherwood J. (2015). A new transcription factor for mitosis: In *Schizosaccharomyces pombe*, the RFX transcription factor Sak1 works with forkhead factors to regulate mitotic expression. Nucleic Acids Res..

[54] Reith W., Mach B. (2001). The bare lymphocyte syndrome and the regulation of MHC expression. Annu. Rev. Immunol..

[55] He S., Song Y., Zhang S., Yuan Z., Yang L., Fang C., Seim I., Liu S., Liu Q., Wang C. (2023). Genetic Innovations Underpin Morphological Diversity and Radiation in Teleost Fish. Res. Sq..

[56] Morotomi-Yano K., Yano K.I., Saito H., Sun Z., Iwama A., Miki Y. (2002). Human regulatory factor X 4 (RFX4) is a testis-specific dimeric DNA-binding protein that cooperates with other humanRFX members. J. Biol. Chem..

[57] Sugiaman-Trapman D., Vitezic M., Jouhilahti E.M., Mathelier A., Lauter G., Misra S., Daub C.O., Kere J., Swoboda P. (2018). Characterization of the human RFX transcription factor family by regulatory and target gene analysis. BMC Genom..

[58] Zhu Z., Younas L., Zhou Q. (2025). Evolution and regulation of animal sex chromosomes. Nat. Rev. Genet..

[59] Guarracino A., Buonaiuto S., de Lima L.G., Potapova T., Rhie A., Koren S., Rubinstein B., Fischer C., Gerton J.L., Human Pangenome Reference Consortium (2023). Recombination between heterologous human acrocentric chromosomes. Nature.

[60] Bertollo L.A.C., Cioffi M.B., Moreira-Filho O., Ozouf-Costaz C., Pisano E., Foresti F., Toledo L.F.A. (2015). Direct chromosome preparation from freshwater teleost fishes. Fish Cytogenetic Techniques (Chondrichthyans and Teleosts).

[61] Sambrook J., Russell D.W. (2001). Molecular Cloning: A Laboratory Manual.

[62] Bolger A.M., Lohse M., Usadel B. (2014). Trimmomatic: A flexible trimmer for Illumina sequence data. Bioinformatics.

[63] Novák P., Ávila Robledillo L., Koblížková A., Vrbová I., Neumann P., Macas J. (2017). TAREAN: A computational tool for identification and characterization of satellite DNA from unassembled short reads. Nucleic Acids Res..

[64] Schmieder R., Edwards R. (2011). Quality control and preprocessing of metagenomic datasets. Bioinformatics.

[65] Smit A., Hubley R., Green P. RepeatMasker Open-4.0. 2013–2015. http://www.repeatmasker.org.

[66] Fu L., Niu B., Zhu Z., Wu S., Li W. (2012). CD-HIT: Accelerated for clustering the next-generation sequencing data. Bioinformatics.

[67] Nascimento M., Sousa A., Ramirez M., Francisco A.P., Carriço J.A., Vaz C. (2017). PHYLOViZ 2.0: Providing scalable data integration and visualization for multiple phylogenetic inference methods. Bioinformatics.

[68] Morgulis A., Coulouris G., Raytselis Y., Madden T.L., Agarwala R., Schäffer A.A. (2008). Database indexing for production MegaBLAST searches. Bioinformatics.

[69] Sassi F.M.C., Toma G.A., Cioffi M.B., Liehr T. (2022). FISH-in fish chromosomes. Cytogenetics and Molecular Cytogenetics.

[70] Sumner A.T. (1972). A simple technique for demonstrating centromeric heterochromatin. Exp. Cell Res..

[71] Goubert C., Modolo L., Vieira C., ValienteMoro C., Mavingui P., Boulesteix M. (2015). De novo assembly and annotation of the Asian tiger mosquito (*Aedes albopictus*) repeatome with dnaPipeTE from raw genomic reads and comparative analysis with the yellow fever mosquito (*Aedes aegypti*). Genome Bio. Evol..

[72] R Core Team (2023). R: A Language and Environment for Statistical Computing.

[73] Eaton D.A. (2014). PyRAD: Assembly of de novo RADseq loci for phylogenetic analyses. Bioinformatics.

[74] Conesa A., Götz S., García-Gómez J.M., Terol J., Talón M., Robles M. (2005). Blast2GO: A universal tool for annotation, visualization and analysis in functional genomics research. Bioinformatics.

[75] Okonechnikov K., Golosova O., Fursov M., Ugene Team (2012). Unipro UGENE: A unified bioinformatics toolkit. Bioinformatics.

