# Supplementary material for: Repeatome Dynamics and Sex Chromosome Differentiation in the XY and XY1Y2 Systems of the Fish Hoplias malabaricus (Teleostei; Characiformes)
Source: Int J Mol Sci. 2025 Jun 24;26(13):6039. doi: 10.3390/ijms26136039 (PMC12250356; doi:10.3390/ijms26136039)
Supplement: Supplementary file 1 [file ijms-26-06039-s001.zip › ijms-3701077-supplementary.pdf]

Supplementary files

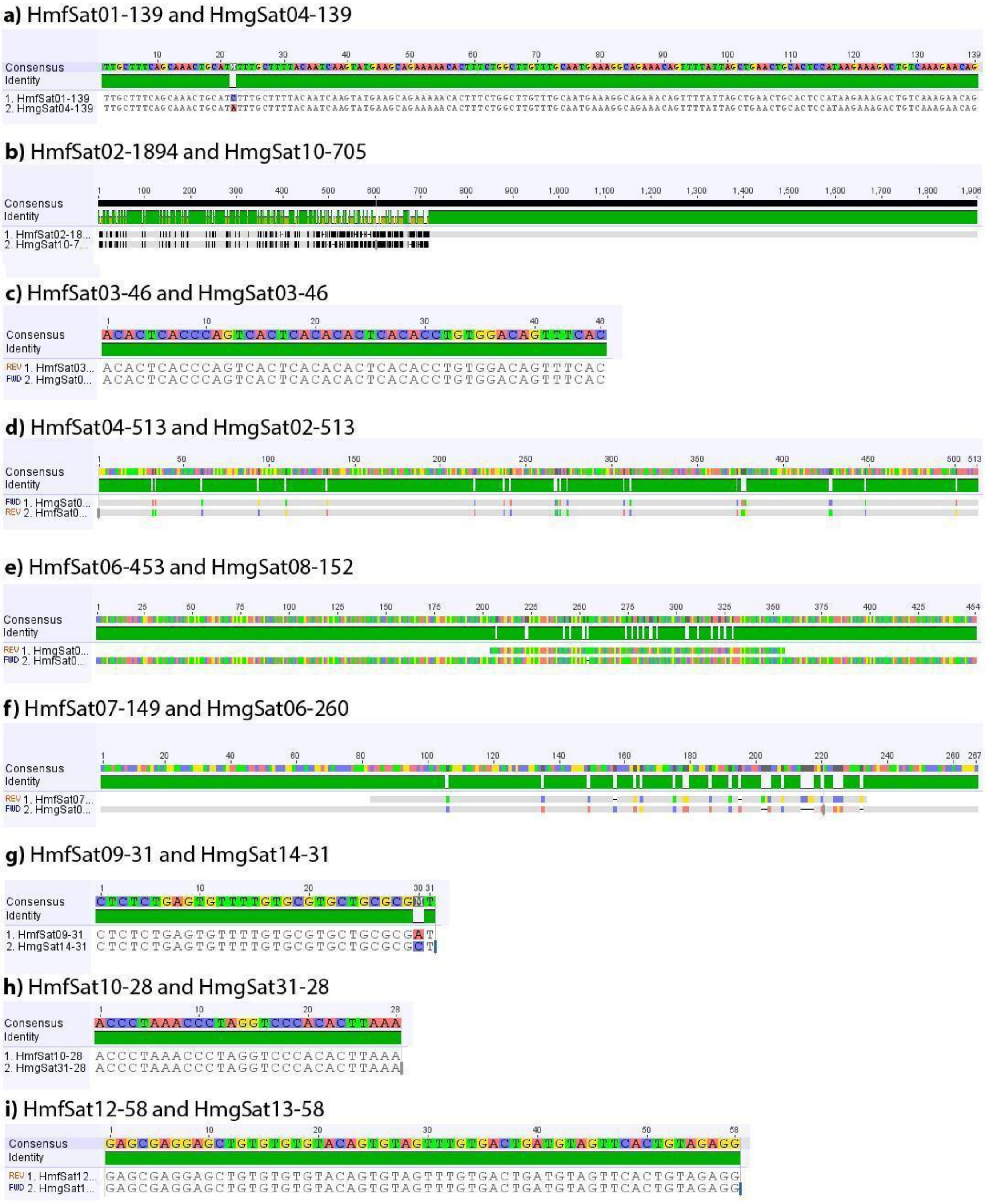

Supplementary Figure S1. Alignments between the conserved satellites of *H. malabaricus* KarF and *H. malabaricus* KarG.

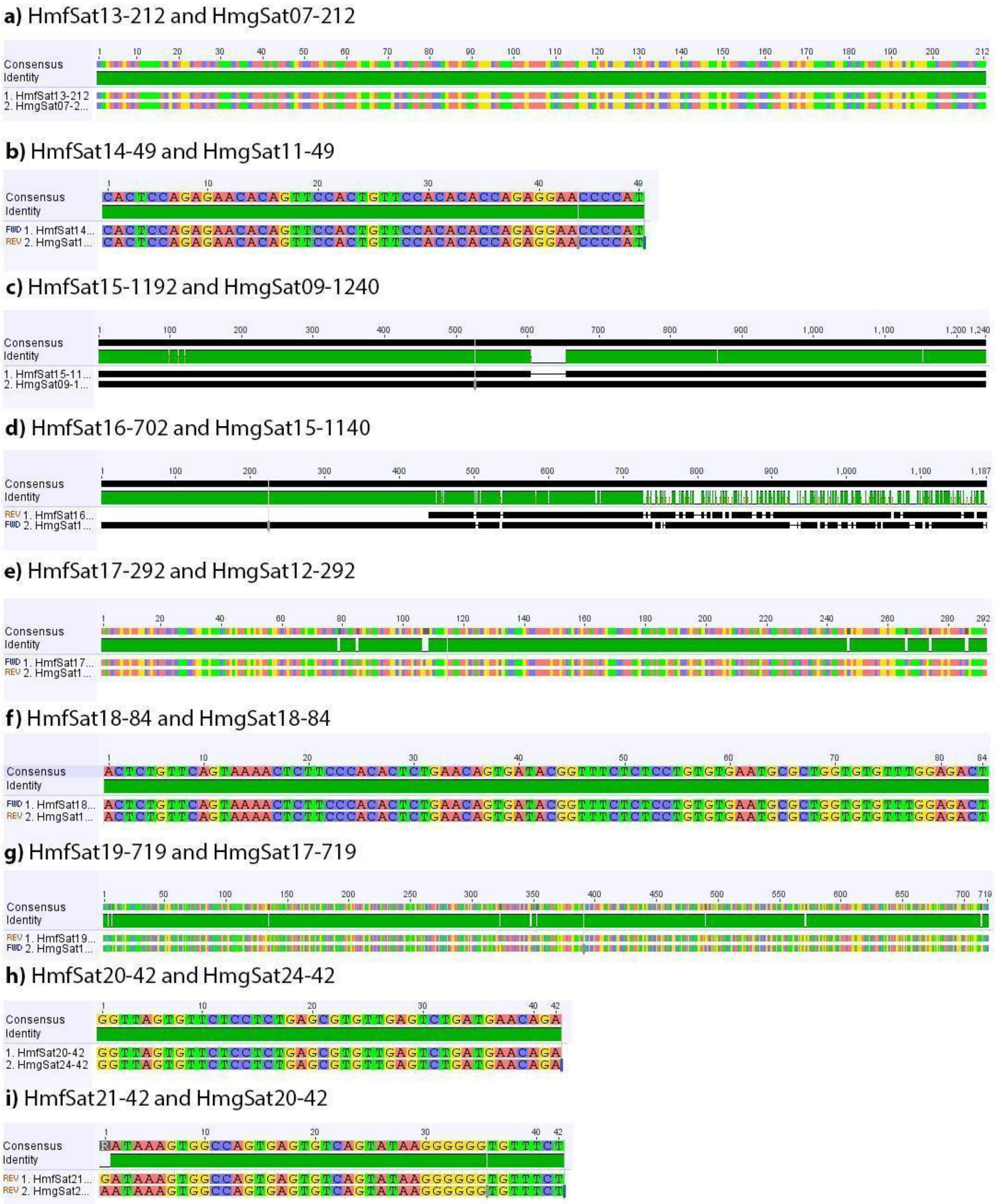

Supplementary Figure S2. Alignments en the conserved satellites of *H. malabaricus* KarF and betwe *H. malabaricus* KarG.

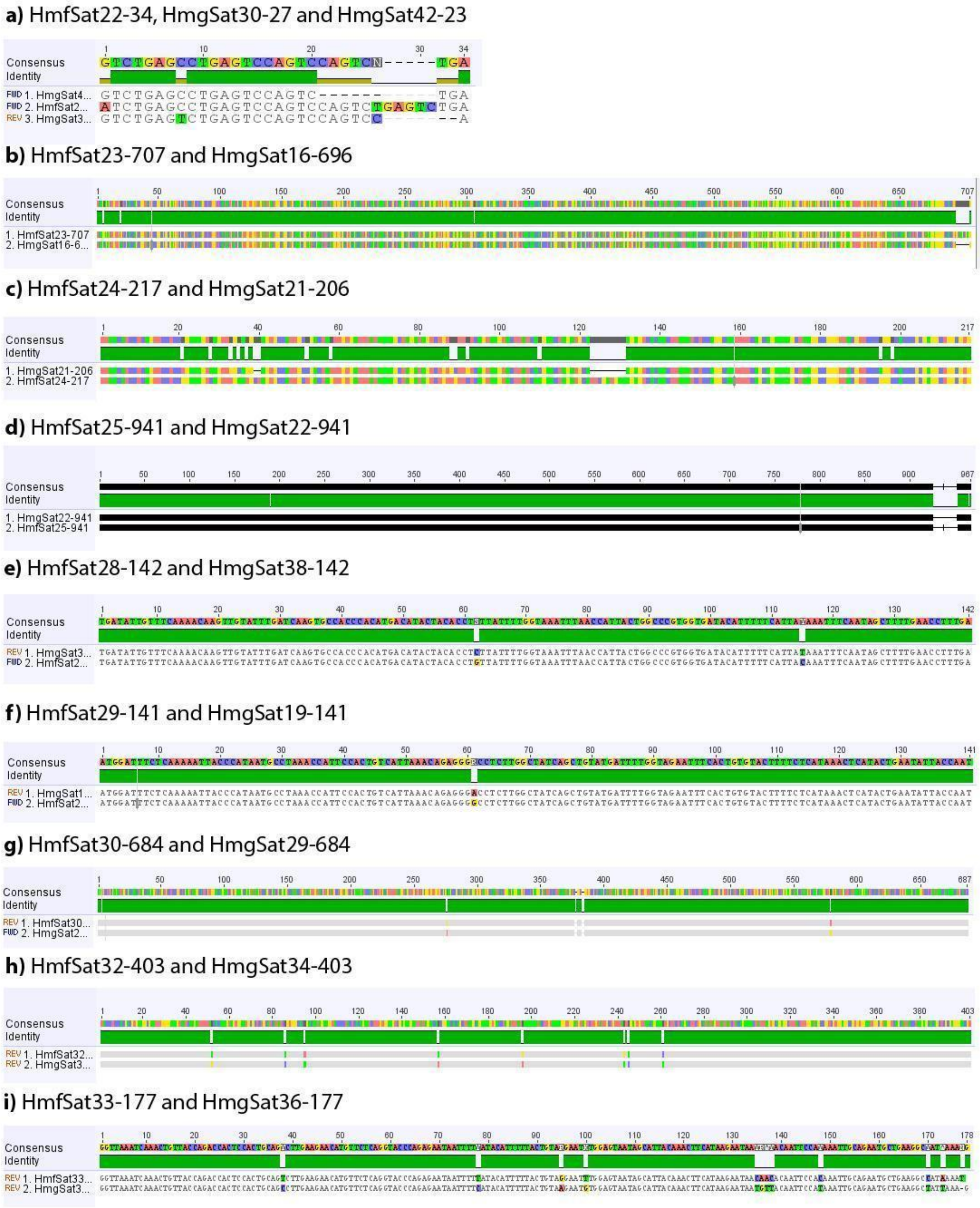

Supplementary Figure S3. Alignments between the conserved satellites of *H. malabaricus* KarF and *H. malabaricus* KarG.

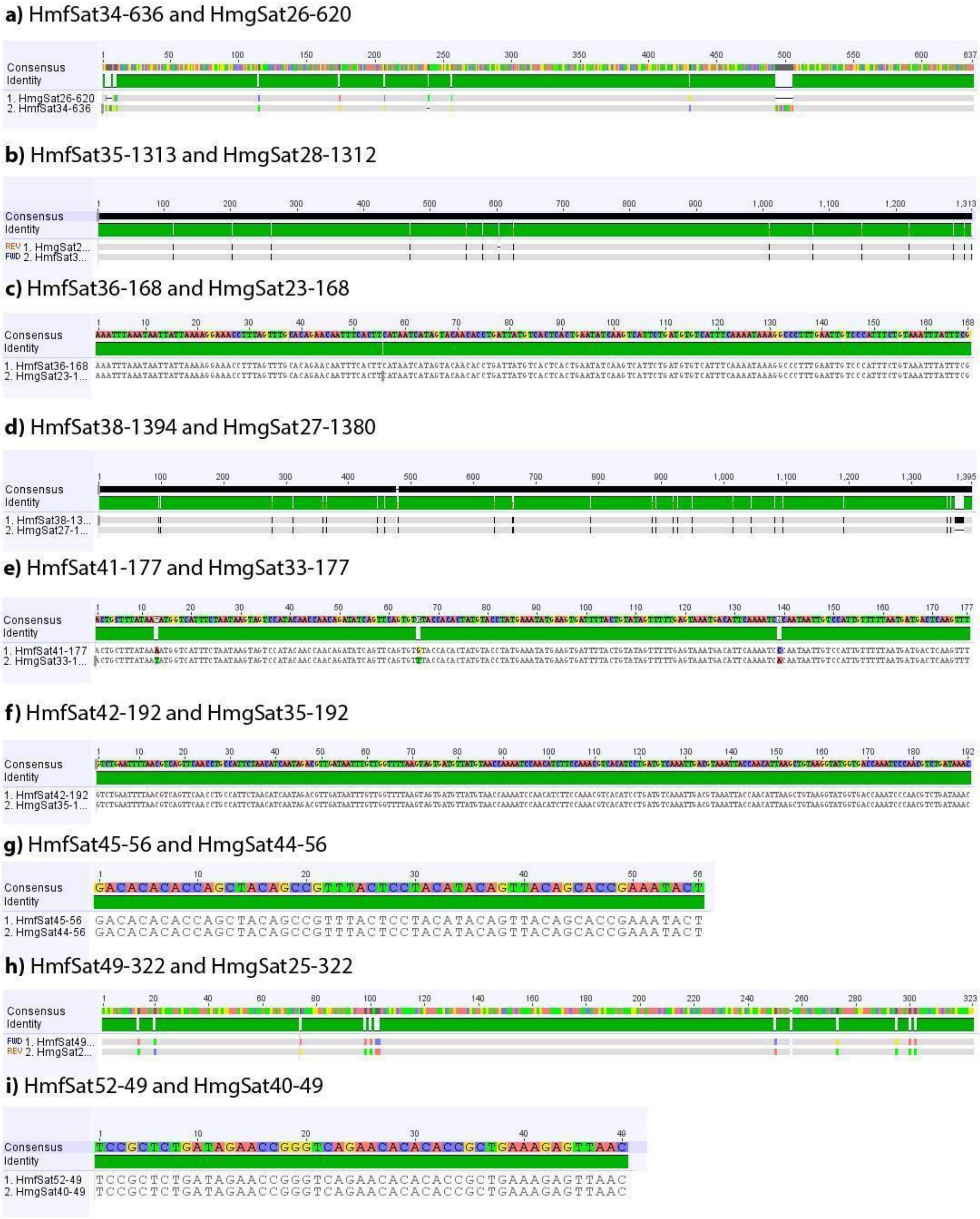

Supplementary Figure S4. Alignments between the conserved satellites of *H. malabaricus* KarF and *H. malabaricus* KarG.

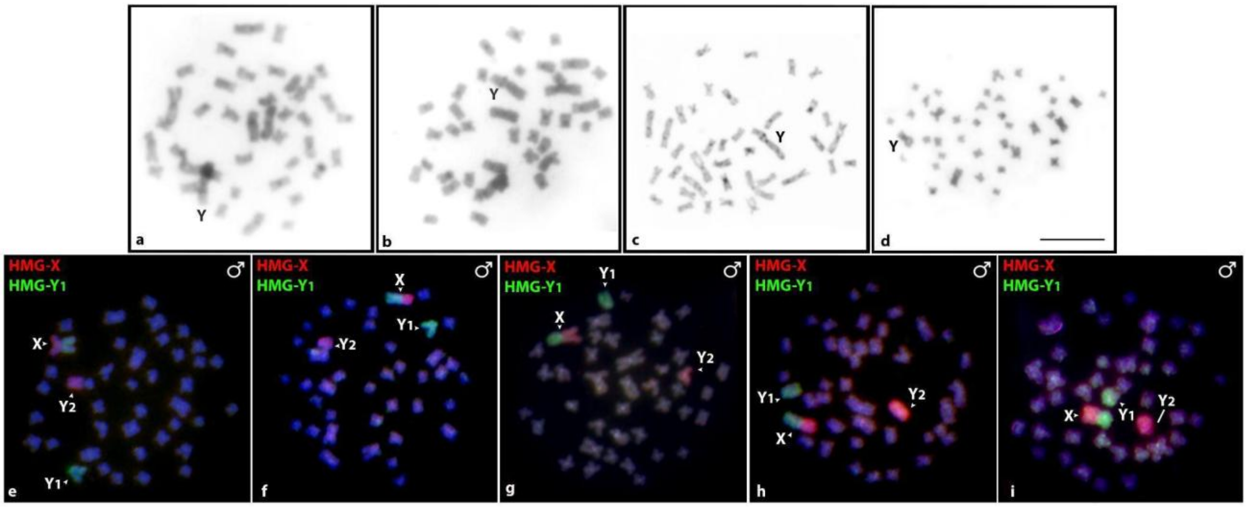

Supplementary Figure S5. Sequential C-banding of the male KarF metaphasic chromosomes extracted from Figure 1 to detect the Y chromosome (a-d) and whole chromosome painting (WCP) of male KarG metaphasic chromosomes using HMG-X and HMG-Y1 probes to detect the sex chromosomes extracted from Figure 2 (e-i).

Supplementary Table S1. General features of *H. malabaricus* KarF satellitome, including the monomer size, the abundance of each satellite in the male and female genomes, abundance in male/female proportion, and divergence.

satDNA family	Monomer size	Abundance male	Abundance female	M/F	Divergence male	Divergence female
HmfSat01-139	139	0.009206394	0.0072728	1.265866525	10.56	10.22
HmfSat02-1894	1894	0.008210824	0.006974806	1.177211787	13.51	13.76
HmfSat03-46	46	0.008194474	0.00947071	0.86524393	8.86	8.58
HmfSat04-513	513	0.006027545	0.005210101	1.156895915	3.78	4.09
*HmfSat05-2936	2936	0.005536974	0.003676283	1.506133778	8.13	11.59
HmfSat06-453	453	0.002245702	0.001872129	1.199544649	3.02	3.14
*HmfSat07-149	149	0.001863612	0.000972741	1.915836445	9.13	9.47
HmfSat08-2944	2944	0.001419167	0.001362754	1.041396211	8.32	8.56
HmfSat09-31	31	0.001339327	0.001310363	1.022103309	10.01	9.69
*HmfSat10-28	28	0.001292539	0.000455365	2.838465034	9.4	8.58
HmfSat11-1922	1922	0.001099455	0.00080017	1.374026816	6.6	7.71
HmfSat12-58	58	0.001007309	0.000989462	1.018036598	2.89	2.86
HmfSat13-212	212	0.000827472	0.000923747	0.895778491	6.09	6.27
HmfSat14-49	49	0.000759121	0.000902111	0.841493878	5.64	5.24
HmfSat15-1192	1192	0.000754676	0.000712374	1.05938168	6.07	5.79
HmfSat16-702	702	0.000701037	0.000669824	1.046598632	1.57	1.62
HmfSat17-292	292	0.000697888	0.000649858	1.073907949	2.61	2.6
HmfSat18-84	84	0.000564623	0.000509023	1.109228905	14.43	14.81
HmfSat19-719	719	0.000530547	0.000502172	1.056503034	1.45	1.65
HmfSat20-42	42	0.000526884	0.000481692	1.093819436	5.64	5.62
HmfSat21-42	42	0.000501426	0.00047726	1.050633463	18.34	18.46
HmfSat22-34	34	0.000488391	0.000422679	1.155465138	13.02	13.07
HmfSat23-707	707	0.000438815	0.000316716	1.385515954	0.81	0.81
HmfSat24-217	217	0.000424977	0.000327981	1.295736594	7.49	7.73
*HmfSat25-941	941	0.000422212	0.00070395	0.599775805	0.91	0.94
*HmfSat26-378	378	0.000396309	0.000202922	1.953013159	12.49	13.97
HmfSat27-53	53	0.000394314	0.000326585	1.207384546	5.28	5
HmfSat28-142	142	0.000391119	0.00039662	0.986130319	7.17	7.27
HmfSat29-141	141	0.000386276	0.000397932	0.970709933	6.59	6.39
HmfSat30-684	684	0.000366284	0.000312424	1.172395325	1.63	1.84
HmfSat31-93	93	0.000311843	0.000323557	0.963793594	5.63	5.57
HmfSat32-403	403	0.00028446	0.000231652	1.227961824	4.25	5.18
HmfSat33-177	177	0.000257428	0.000196419	1.310606252	13.84	15.4
HmfSat34-636	636	0.000240682	0.000243166	0.989784119	4.28	3.74
HmfSat35-1313	1313	0.000239686	0.000209203	1.145710973	3.19	3.32
HmfSat36-168	168	0.00022959	0.00023432	0.97981518	7.96	7.86
*HmfSat37-841	841	0.000222987	0.000143061	1.558681449	10.46	10.9
*HmfSat38-1394	1394	0.00022106	0.000268491	0.823343511	1.29	1.17
HmfSat39-57	57	0.000208419	0.000210881	0.988323395	1.71	1.53
HmfSat40-457	457	0.00020737	0.000195332	1.06163165	6.92	7.48
HmfSat41-177	177	0.00020577	0.000230017	0.894587998	11	11.06
HmfSat42-192	192	0.000178224	0.00020592	0.865500844	5	4.64
HmfSat43-129	129	0.000169116	0.000185508	0.911636769	5.61	5.78
HmfSat44-186	186	0.00016579	0.000177982	0.931498314	2.09	2.02
HmfSat45-56	56	0.000163946	0.000160292	1.022792551	4.67	4.93
*HmfSat46-50	50	0.000154555	5.25E-05	2.945968899	1.45	1.3
*HmfSat47-326	326	0.000149251	4.89E-05	3.054465878	2.86	6.85

HmfSat48-42	42	0.000137447	0.000102664	1.338795074	4.73	5.16
*HmfSat49-322	322	0.000136745	0.000183475	0.745302466	5.57	5.64
HmfSat50-199	199	0.000125397	0.000139625	0.898100283	4.82	5.06
HmfSat51-83	83	0.000115893	0.000123792	0.936191314	1.86	1.37
HmfSat52-49	49	0.00011306	9.67E-05	1.169187136	6.2	5.95
HmfSat53-41	41	0.000111406	0.000172385	0.646261824	4.78	4.58
HmfSat54-35	35	9.17E-05	8.71E-05	1.05285172	4.57	4.57
HmfSat55-47	47	8.07E-05	7.29E-05	1.107707362	7.8	7.56
HmfSat56-126	126	7.03E-05	7.15E-05	0.983124991	9.66	9.74

Supplementary Table S2. General features of *H. malabaricus* KarG satellitome, including the monomer size, the abundance of each satellite in the male and female genomes, abundance in male/female proportion, and divergence.

satDNA family	Monomer size	Abundance male	Abundance female	F/M	Divergence male	Divergence female
HmgSat01-58	58	0.02625331	0.027450575	0.95638469	2.95	2.9
*HmgSat02-513	513	0.01583843	0.01049602	1.508993885	3.87	3.91
HmgSat03-46	46	0.007306839	0.006170622	1.184133281	9.49	9.51
HmgSat04-139	139	0.004704228	0.004969837	0.946555806	12.56	12.44
*HmgSat05-63	63	0.003327413	0.002287971	1.454307093	6.79	6.81
HmgSat06-260	260	0.002008794	0.002229842	0.900868507	13.24	11.36
HmgSat07-212	212	0.002041201	0.001908067	1.069774091	6.12	6.47
HmgSat08-152	152	0.002022685	0.001585596	1.275662336	15.88	15.85
HmgSat09-1240	1240	0.000803655	0.000874338	0.919159171	8.98	10.46
HmgSat10-705	705	0.000927718	0.000862115	1.076095395	16.24	16.09
HmgSat11-49	49	0.000729399	0.00076433	0.95429892	5.3	5.22
HmgSat12-292	292	0.00089935	0.000702072	1.280992761	2.31	2.32
HmgSat13-58	58	0.00069729	0.000687842	1.013736469	2.83	2.91
HmgSat14-31	31	0.000813639	0.000645255	1.260955827	9.07	9.18
HmgSat15-1140	1140	0.000694786	0.000576824	1.204503323	2.35	2.25
HmgSat16-696	696	0.000646487	0.000564546	1.1451461	1.06	1.07
HmgSat17-719	719	0.000525651	0.00054403	0.966218356	1.35	1.32
HmgSat18-84	84	0.000422373	0.000504293	0.83755517	14.83	15.29
HmgSat19-141	141	0.000538781	0.000461919	1.166398021	5.98	5.91
HmgSat20-42	42	0.000513434	0.00045691	1.123708771	17.76	18.28
*HmgSat21-206	206	0.000302542	0.000445914	0.678475397	7.58	7.04
HmgSat22-941	941	0.000373557	0.00035215	1.060789154	0.77	0.73
HmgSat23-168	168	0.000311697	0.000329272	0.946623968	7.71	7.34
HmgSat24-42	42	0.000326573	0.000304269	1.073303353	6.26	5.77
HmgSat25-322	322	0.000256329	0.00029605	0.865830574	4.81	4.37
HmgSat26-620	620	0.000242262	0.00027497	0.881049258	3.2	2.9
HmgSat27-1380	1380	0.000333226	0.000271407	1.227771677	0.7	0.74
*HmgSat28-1312	1312	0.000209642	0.000270847	0.774023505	1.24	1.42
HmgSat29-684	684	0.000243473	0.000265859	0.915797079	1.1	1.06
HmgSat30-27	27	0.00028416	0.000248715	1.142514789	10.77	10.86
HmgSat31-28	28	0.000342228	0.000235337	1.454205057	8.63	8.3
*HmgSat32-827	827	0.000150596	0.00020717	0.72691907	2.06	2.07
HmgSat33-177	177	0.000237331	0.000203107	1.168501204	10.9	11.09
HmgSat34-403	403	0.000201965	0.000191261	1.055965088	4.78	5.93
HmgSat35-192	192	0.000227919	0.000186896	1.219495139	5.85	6.01
HmgSat36-177	177	0.00017485	0.000173289	1.009010296	15.72	16.05
*HmgSat37-467	467	0.000263549	0.000173259	1.521120966	8.76	9.41
HmgSat38-142	142	0.000155708	0.0001542	1.009778993	7.86	8.01
HmgSat39-34	34	0.000113989	0.000153018	0.744940083	8.48	7.88
HmgSat40-49	49	0.000143369	0.000144479	0.992317919	5.97	5.82
HmgSat41-21	21	0.000147602	0.000140705	1.049017676	7.08	6.76
HmgSat42-23	23	0.000151921	0.000133235	1.140249838	14.83	14.83
HmgSat43-142	142	0.000169662	0.000129129	1.313901242	6.04	5.96
HmgSat44-56	56	0.000127727	0.000115051	1.110170221	6.12	6.17
HmgSat45-588	588	9.81E-05	0.000114585	8.56E-01	13.51	12.67

Supplementary Table S3. Shared satDNAs between karyomorphs F and G of *H. malabaricus*.

satDNA family (Hmf)	satDNA family (Hmg)	Similarity (%)
HmfSat01-139	HmgSat04-139	99.28
HmfSat02-1894	HmgSat10-705	74.51
HmfSat03-46	HmgSat03-46	100
HmfSat04-513	HmgSat02-513	95.52
HmfSat06-453	HmgSat08-152	83.55
HmfSat07-149	HmgSat06-260	83.44
HmfSat09-31	HmgSat14-31	96.77
HmfSat10-28	HmgSat31-28	100
HmfSat12-58	HmgSat13-58	100
HmfSat13-212	HmgSat07-212	100
HmfSat14-49	HmgSat11-49	100
HmfSat15-1192	HmgSat09-1240	95.72
HmfSat16-702	HmgSat15-1140	67.38
HmfSat17-292	HmgSat12-292	97.26
HmfSat18-84	HmgSat18-84	100
HmfSat19-719	HmgSat17-719	98.75
HmfSat20-42	HmgSat24-42	100
HmfSat21-42	HmgSat20-42	97.62
HmfSat22-34	HmgSat30-27/ HmgSat42-23	70.58/68.96
HmfSat23-707	HmgSat16-696	98.02
HmfSat24-217	HmgSat21-206	87.67
HmfSat25-941	HmgSat22-941	99.57
HmfSat28-142	HmgSat38-142	98.59
HmfSat29-141	HmgSat19-141	99.29
HmfSat30-684	HmgSat29-684	99.70
HmfSat32-403	HmgSat34-403	98.01
HmfSat33-177	HmgSat36-177	92.69
HmfSat34-636	HmgSat26-620	95.76
HmfSat35-1313	HmgSat28-1312	98.85
HmfSat36-168	HmgSat23-168	100
HmfSat38-1394	HmgSat27-1380	97.05
HmfSat41-177	HmgSat33-177	98.30
HmfSat42-192	HmgSat35-192	100
HmfSat45-56	HmgSat44-56	100
HmfSat49-322	HmgSat25-322	96.27
HmfSat52-49	HmgSat40-49	100

Supplementary Table S4. DNApipeTE detailed repeat classification. All values are presented in percentages (%) of each genome depicted above with distinct coverages. A graphical representation is provided in **Figure 5** for better visualization.

	**KarF Female**				**KarF Male**				**KarG Female**				**KarG Male**			
	**0.1×**	**0.25×**	**0.5×**	**Average**	**0.1×**	**0.25×**	**0.5×**	**Average**	**0.1×**	**0.25×**	**0.5×**	**Average**	**0.1×**	**0.25×**	**0.5×**	**Average**
LTR	1,6	1,66	2,31	1,86	1,21	1,51	2,31	1,68	0,85	1,63	2,02	1,5	1,99	1,83	2,44	2,0866
LINE	2,64	3,42	3,72	3,26	2,59	2,97	3,46	3,01	2,51	3,25	3,87	3,21	2,4	2,98	3,6	2,9933
SINE	0,33	0,39	0,53	0,42	0,59	0,39	0,54	0,51	0,37	0,56	0,73	0,55	0,39	0,45	0,66	0,5
DNA transposons	7,89	9,99	12,79	10,22	7,14	10,37	11,96	9,82	9,06	10,69	16,65	12,13	7,33	10,42	13,19	10,3133
Helitron	2,72	3,31	2,68	2,90	2,57	1,7	3,18	2,48	0,54	1,58	1,01	1,04	1,32	1,97	1,75	1,68
rRNA	0,87	1,25	0,96	1,03	0,92	0,95	1,34	1,07	0,42	0,62	0,69	0,58	1	0,85	0,81	0,886
Low Complexity	0,87	0,51	1,11	0,83	1,1	0,70	1,12	0,97	0,29	0,25	0,52	0,35	0,41	0,65	0,78	0,613
Satellite	0,04	0,11	0,15	0,10	0,06	0,06	0,15	0,09	0,03	0,22	0,16	0,14	0,12	0,1	0,13	0,11
Simple repeat	0,79	1,51	1,97	1,42	0,69	1,19	1,97	1,28	0,68	1,21	1,55	1,15	0,79	0,86	1,85	1,16
Others	0,33	0,43	0,54	0,43	0,38	0,49	0,61	0,49	0,44	0,53	0,56	0,51	0,45	0,52	0,54	0,50
Na	12,87	13,58	21,57	16,01	13,82	16,55	22,31	13,08	13,05	13,8	23,29	16,71	12,57	14,5	22,97	16,68
**Repetitive total**	30,95	36,16	48,33	38,47	31,07	36,88	48,95	34,48	28,24	34,34	51,05	37,87	28,77	35,13	48,72	37,54
Single or low copy DNA	69,49	63,85	51,67	61,67	68,93	63,13	51,06	61,04	71,77	65,65	51,95	63,12	71,22	64,85	51,27	62,44

Supplementary Table S5. Paired t-test for the averages of repetitive content recovered by DNApipeTE. Sex is represented by F for females and M for males.

**Karyomorph**	**Sex**	**T-test (p-value)**
F	F	0.198
F	M	
G	F	0.880
G	M	
F	F	0.580
G	F	
F	M	0.444
G	M	

Supplementary Table S6. Putative sex-linked markers obtained from DArT-Seq, according to each *Hoplias malabaricus* karyomorphs (K), its sequence, BLAST result (BLAST), Length of the sequence, e-value from BLAST, mean of similarity between query and hit (sim mean), and the indication (Sex) of male- (XY) or female-heterogametic sex system (ZW).

**K**	**Seq**	**Length**	**BLAST**	**e-value**	**Sim Mean**	**Sex**
G	TGCAGAATAGCTACACTGGCTCCCAGAGAGCCAACCAGGACCTGGAGGAGAAGCTGCATG	60	*Scleropages formosus* tight junction associated protein 1 (tjap1), transcript variant X8, mRNA	2,72E-17	94,2	XY
G	TGCAGGGGCACCACGGTTTGGCTTACCTTGTCCTATGCATGGGCCAACTTCAGCATG	57	*Scleropages formosus* type-1 angiotensin II receptor-associated protein-like (LOC108933497), mRNA	6,34E-18	98,11	XY
G	TGCAGGACGGTGGGCATAAGCACCCCAGAGATTGCTTTGTACAGGATGGAGTCACAGACACCCACGATG	69	*Scleropages formosus* regulatory factor X4 (rfx4), mRNA	5,84E-25	93,03	XY
G	TGCAGGAACGTACCAGGGCCAGTGGGTGGGGGGCATG	37	*Scleropages formosus* junctophilin 3 (jph3), transcript variant X4, mRNA	6,53E-07	97,01	XY

G	TGCAGAATGCATTTTAAAATGTATGCAATAGTACAGTGTATTTCACGATGCATG	54	*Scleropages formosus* traf2 and NCK-interacting protein kinase-like (LOC108927730), transcript variant X6, mRNA	5,20E-09	90,74	XY
G	TGCAGCAAGAAGAAGAGAAGCGGCGCTTGGGCCGTGAAGCTAGCATTATCACAGCCATCCCTCTCACTC	69	*Scleropages formosus* unc-80 homolog, NALCN channel complex subunit (unc80), transcript variant X12, mRNA	1,32E-16	93,08	XY
G	TGCAGGTTATCATGGCCATGCCGGCTGTCAGGTTGCGCATCATGTGGTGAGCCGCTACTCGCATG	65	*Scleropages formosus* CCR4-NOT transcription complex subunit 1 (LOC108926306), transcript variant X2, mRNA	3,18E-20	96,02	XY
G	TGCAGGGGACATGGTGGAGATTGTGGAGAAGAATCCAAATGGTGAGCATG	50	*Trichomycterus rosablanca* neutrophil cytosolic factor 1 (ncf1), mRNA	1,11E-06	92,86	XY
G	TGCAGAGATGACAGGGGGAAAGCTTTATTGCTCCGAGTTTGTATCGATCGGGGCCCTTCACAGGGGGCA	69	*Trachurus japonicus* A0001 DNA, chromosome 16, sequence	5,62E-22	97,16	XY
G	TGCAGGTACAGGAACATCAACCGCAAGCTGTCCGCCGCCAGCATG	45	*Brienomyrus brachyistius* 5-hydroxytryptamine receptor 7-like (LOC125715725), mRNA	1,07E-15	96,97	XY

G	TGCAGAGAGACCTCCAGGAAGTAGTGTGTGTACTTGTCCATGAAAGCTTTGGTCTTGGAAAGGGAAGGC	69	*Scleropages formosus* cyclin J like (ccnjl), transcript variant X2, mRNA	9,83E-30	95,92	XY
G	TGCAGCTTGAAGCACTTGCCGCAGATGGGGCAGGCGTGTGGGCGCACGTTGGAATGGACCAGGCGATGC	69	*Scleropages formosus* zinc finger protein 13-like (LOC108919821), mRNA	3,47E-20	96,32	XY
G	TGCAGCTCCTGTCATGTCGCATTTGCATAGGAGAGGATGGATAACCAAATCAGCGCGCCGAGGAAGTCG	69	*Conger conger* RNA binding motif protein 26 (rbm26), mRNA	3,22E-14	91,33	ZW
G	TGCAGTCCAGTGGAATAGCATG	22	NA	NA	NA	XY
G	TGCAGACGTTATGTCATGGTGTCACGGCCCACTGAAAAGCAACAGTAGCCCTCATCTACATGAGCACAC	69	NA	NA	NA	XY
G	TGCAGAGGGAGCACGAGCAGTTCCACATG	29	NA	NA	NA	XY
G	TGCAGATGCTGCTAAACCCTGAGCAGGGCAGCCTGGCACTGCATG	45	NA	NA	NA	XY
G	TGCAGGTGGTCTTCGGAACATG	22	NA	NA	NA	XY
G	TGCAGATTTCTGGATCTTTCCCCAGCTCCTTCCCGGCAGTGAGGCATG	48	NA	NA	NA	XY
G	TGCAGTGGAACTGCCTCTGCATG	23	NA	NA	NA	XY
G	TGCAGCTGCAAACACTGTGCTGCATG	26	NA	NA	NA	XY

G	TGCAGATCAGTGACTTTGAGCATG	24	NA	NA	NA	XY
G	TGCAGCACCCCCGTGTCCCCACTCCACATG	30	NA	NA	NA	XY
G	TGCAGTGGCGCAGGGGCTGCGCAGCGACACCCTCAGAGTACCCAGGCATG	50	NA	NA	NA	XY
G	TGCAGTTGCGTCATTGGAATGTCGTCATG	29	NA	NA	NA	XY
G	TGCAGCCGCCCCACCCGTATGCATG	25	NA	NA	NA	XY
G	TGCAGTTGTCATGGTGCTGTTCTGATGCTTTCTGTTGGTATCCAGATGGAGAAGCATG	58	NA	NA	NA	XY
G	TGCAGCCTGCTGTATTTCATG	21	NA	NA	NA	XY
G	TGCAGAACTGGCACCACTGGCATG	24	NA	NA	NA	XY
G	TGCAGGGGGCAGGGGCCCAGACATG	25	NA	NA	NA	XY
G	TGCAGATTGATGGCTCCACCCTGCGGACCCTGTCATG	37	NA	NA	NA	XY
G	TGCAGAGAGCATAGTATGCAAGCATG	26	NA	NA	NA	XY
G	TGCAGCACCGCACCTGCCCCCTATCATG	28	NA	NA	NA	XY
G	TGCAGGAACCGCACAAACATG	21	NA	NA	NA	XY
G	TGCAGCTGTGGAAATTTGATGCATG	25	NA	NA	NA	XY
G	TGCAGGACCTGCGTCGGCATG	21	NA	NA	NA	XY

G	TGCAGATGAAAAGCCTGAGCTACGCTTGCCAGGCTCTGGTTCCTACTGAGCATG	54	NA	NA	NA	XY
G	TGCAGAATGACAGGAACGCATG	22	NA	NA	NA	XY
G	TGCAGGACTACTACCAAAGCGGACGCATG	29	NA	NA	NA	XY
G	TGCAGCTTTTCTGAAATTTCAGCATG	26	NA	NA	NA	XY
G	TGCAGACCATACTGCGCATG	20	NA	NA	NA	XY
G	TGCAGGAAGAGATAAGGGACAGGAAAGTGTTCTCTGCATG	40	NA	NA	NA	XY
G	TGCAGCAGCAGCAAGCTTCCAGTTAGCATG	30	NA	NA	NA	XY
G	TGCAGTGGGTGCAGTTAATGCATG	24	NA	NA	NA	XY
G	TGCAGCTTTGATTAATTGTCCACATG	26	NA	NA	NA	XY
G	TGCAGCATAAACATAGTTTGAATATTTTATGTGTGTATCATGCATG	46	NA	NA	NA	XY
G	TGCAGAGAGTAACAAGAGACACACTTCACAGTCACTACTCAACCAGGACACCAGTGGGCACATGCAAAA	69	NA	NA	NA	XY
G	TGCAGCTTCTCCGTATCCCACATG	24	NA	NA	NA	XY
G	TGCAGATTCGAGTGGACACCAAGAAAGCATG	31	NA	NA	NA	XY
G	TGCAGAGTCCTACATCTGTCAAAGAGAAGCTCCTCCGCCTGTGGATACAACGCTGTAAGATTTACAGTC	69	NA	NA	NA	XY
